# Expression and Role of Gonadotropin-Releasing Hormone 2 and Its Receptor in Mammals

**DOI:** 10.3389/fendo.2017.00269

**Published:** 2017-12-11

**Authors:** Amy T. Desaulniers, Rebecca A. Cederberg, Clay A. Lents, Brett R. White

**Affiliations:** ^1^Department of Animal Science, University of Nebraska-Lincoln, Lincoln, NE, United States; ^2^USDA, ARS, USMARC, Clay Center, NE, United States

**Keywords:** GnRH2, GnRH2 receptor, reproductive function, G protein-coupled receptor, G protein-coupled receptor signal transduction, autocrine/paracrine mechanisms, testis, cancer

## Abstract

Gonadotropin-releasing hormone 1 (GnRH1) and its receptor (GnRHR1) drive mammalian reproduction *via* regulation of the gonadotropins. Yet, a second form of GnRH (GnRH2) and its receptor (GnRHR2) also exist in mammals. GnRH2 has been completely conserved throughout 500 million years of evolution, signifying high selection pressure and a critical biological role. However, the *GnRH2* gene is absent (e.g., rat) or inactivated (e.g., cow and sheep) in some species but retained in others (e.g., human, horse, and pig). Likewise, many species (e.g., human, chimpanzee, cow, and sheep) retain the *GnRHR2* gene but lack the appropriate coding sequence to produce a full-length protein due to gene coding errors; although production of GnRHR2 in humans remains controversial. Certain mammals lack the *GnRHR2* gene (e.g., mouse) or most exons entirely (e.g., rat). In contrast, old world monkeys, musk shrews, and pigs maintain the coding sequence required to produce a functional GnRHR2. Like GnRHR1, GnRHR2 is a 7-transmembrane, G protein-coupled receptor that interacts with G_αq/11_ to mediate cell signaling. However, GnRHR2 retains a cytoplasmic tail and is only 40% homologous to GnRHR1. A role for GnRH2 and its receptor in mammals has been elusive, likely because common laboratory models lack both the ligand and receptor. Uniquely, both GnRH2 and GnRHR2 are ubiquitously expressed; transcript levels are abundant in peripheral tissues and scarcely found in regions of the brain associated with gonadotropin secretion, suggesting a divergent role from GnRH1/GnRHR1. Indeed, GnRH2 and its receptor are not physiological modulators of gonadotropin secretion in mammals. Instead, GnRH2 and GnRHR2 coordinate the interaction between nutritional status and sexual behavior in the female brain. Within peripheral tissues, GnRH2 and its receptor are novel regulators of reproductive organs. GnRH2 and GnRHR2 directly stimulate steroidogenesis within the porcine testis. In the female, GnRH2 and its receptor may help mediate placental function, implantation, and ovarian steroidogenesis. Furthermore, both the *GnRH2* and *GnRHR2* genes are expressed in human reproductive tumors and represent emerging targets for cancer treatment. Thus, GnRH2 and GnRHR2 have diverse functions in mammals which remain largely unexplored.

## Background

### The Classical Form of Mammalian Gonadotropin-Releasing Hormone (GnRH1)

The classical, hypophysiotropic GnRH1 is hailed as the master regulator of reproduction in mammals. GnRH1 is a decapeptide (pGlu–His–Trp–Ser–Tyr–Gly–Leu–Arg–Pro–Gly–NH_2_) produced by hypothalamic neurons and secreted in a pulsatile manner into hypophyseal portal capillaries where it travels to the anterior pituitary gland. GnRH1 then binds to its receptor (GnRHR1) on gonadotrope cells, promoting the synthesis and secretion of the gonadotropins, follicle-stimulating hormone (FSH) and luteinizing hormone (LH), into peripheral circulation where they act on their target organs, the gonads. In females, FSH stimulates follicular development, whereas LH promotes ovulation and maintenance of the corpus luteum. Within the testes, FSH regulates spermatogenesis and LH elicits secretion of testosterone. Ultimately, the gonads cease to function and reproduction is halted in the absence of GnRH1 (1–3).

### GnRH Variants in Mammals

Gonadotropin-releasing hormone 1 was first identified in the hypothalami of pigs and sheep (4–6) and was originally thought to be a novel peptide. However, 23 other forms of GnRH have since been discovered (7), all with 10 amino acids and at least a 50% sequence identity (8). Within these forms, the sequences of both the N-terminus (pGlu–His–Trp–Ser) and C-terminus (Pro–Gly–NH_2_) are conserved (7, 9). The amino acid substitutions only occur between residues 5 and 8 (7, 9). In vertebrates, three forms of GnRH (GnRH1, GnRH2, and GnRH3) are the most common. The third form of GnRH (GnRH3; pGlu–His–Trp–Ser–His–Asp–Trp–Lys–Pro–Gly–NH_2_) was first discovered in lamprey (10) but the *GnRH3* gene has only been confirmed in fish and amphibians to date (7, 11). Therefore, only GnRH1 and GnRH2 are produced in mammals (7).

## Gonadotropin-Releasing Hormone 2

### The Second Form of Mammalian GnRH (GnRH2)

A second structural variant of GnRH, GnRH2, has been identified in mammals. Like GnRH1, GnRH2 is a decapeptide but it was first isolated from the hypothalami of 10,000 chickens and therefore named “chicken GnRH2” (12). It was later discovered in mammals, the first being marsupials (13), and renamed simply “GnRH2” to prevent confusion (14). Since then, GnRH2 has been found in animals of every vertebrate class including primitive orders (e.g., bony fish) as well as complex mammals (15). GnRH2 is absent only in jawless fish (16). Notably, the sequence of GnRH2 remains entirely conserved throughout evolution, indicating high selection pressure and a critical function (17). Absolute conservation of GnRH2 has persisted despite 500 million years of evolution (15), indicating that it may be the most ancient form of GnRH (18). In contrast, GnRH1 evolved 350 million years ago and its sequence varies greatly among vertebrates (19).

### The Gene for GnRH2

**Figure 1:**
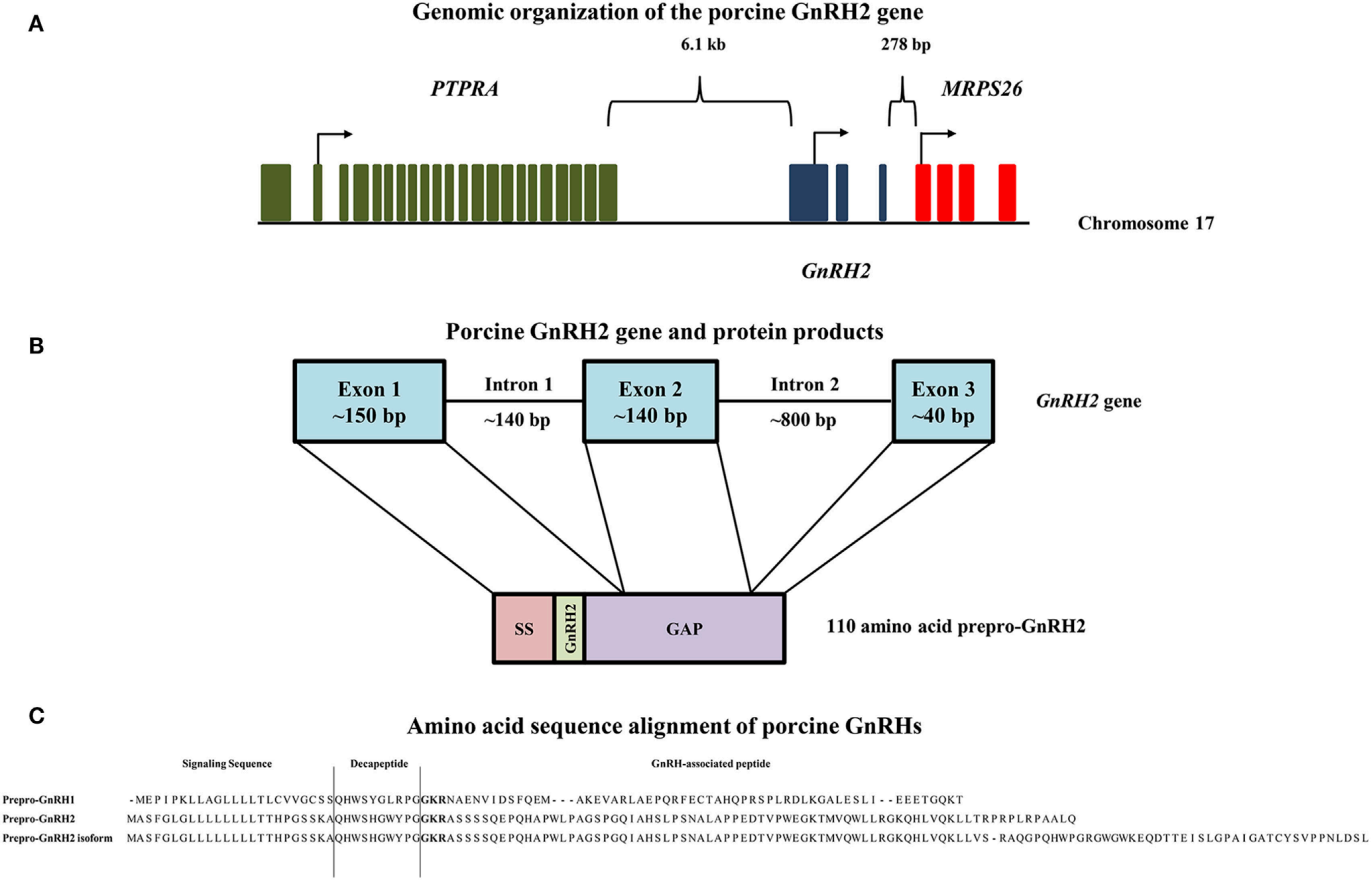
Genomic organization of the porcine GnRH2 gene and its products. **(A)** The *PTPRA* gene (green) is located 6.1 kb upstream, whereas the *MRPS26* gene (red) is positioned 278 bp downstream of the porcine *GnRH2* gene (blue) on chromosome 17. Arrows indicate start codons for each gene. **(B)** The porcine *GnRH2* gene contains three coding exons and two introns. Exon 1 (~150 bp) of the porcine *GnRH2* gene encodes the signaling sequence (SS), mature GnRH2 decapeptide, and a portion of the GnRH-associated peptide (GAP). Exon 2 (~140 bp) and exon 3 (~40 bp) encode the remaining GAP sequence. Note that introns and exons are not drawn to scale. **(C)** Amino acid sequence alignments of predicted porcine prepro-GnRH2 isoforms (NCBI accession numbers XP_005672842 and XP_013840618) with porcine prepro-GnRH1 (NCBI accession number NP_999439). Prepro-GnRH1 is 91 amino acids in length compared with prepro-GnRH2, which has 110 residues. An isoform of prepro-GnRH2 (143 amino acids) is also predicted to be produced from the porcine *GnRH2* gene due to alternative splicing. The amino acids that correspond to the SS, the mature decapeptide, and the GAP are indicated. The proteolytic cleavage sites are highlighted in bold.

GnRH2 is not merely a splice variant of the *GnRH1* gene; instead, it is produced from its own gene that encodes the peptide, prepro-GnRH2 (20). The *GnRH2* gene is located on chromosome 20 in humans, chimpanzees, and orangutans, chromosome 13 in the cow, chromosome 22 in the horse, chromosome 10 in the rhesus macaque, and chromosome 17 in the pig (21). The genomic orientation of the *GnRH2* gene is highly conserved across species (21, 22). It is flanked by the *PTPRA* and *MRPS26* genes in all mammalian and non-mammalian vertebrates examined to date (21, 22). The *PTPRA* gene resides about 5–6 kb upstream of the *GnRH2* gene (21) and encodes the enzyme, receptor-type tyrosine-protein phosphatase α, which is critical for neural development (23). The *MRPS26* gene resides about 300 bp downstream of the *GnRH2* gene (21), encoding mitochondrial ribosome protein S26, which assists in protein synthesis (24). A graphical representation of the porcine *GnRH2* gene is depicted in Figure 1A.

The human *GnRH2* gene has three coding exons like the *GnRH1* gene; however, the *GnRH2* gene is notably shorter (2.1 versus 5.1 kb), primarily due to differences in intron length (20). Otherwise, organization of the *GnRH1* and *GnRH2* genes remain similar (25). The first coding exon in humans encodes the signal sequence, mature decapeptide, and a portion of the GnRH-associated peptide (GAP). The second and third exons encode the remaining GAP (20). Likewise, porcine prepro-GnRH2 is encoded by 3 exons and yields a 110 amino acid product (Figure 1B) that must undergo post-translational proteolytic processing for functionality (20).

### Presence of the GnRH2 Gene in Mammals

Although the *GnRH2* gene was first identified in humans (20), Stewart et al. (21) examined the genomes of mammals encompassing 10 orders for the presence of the *GnRH2* gene. The *GnRH2* gene was positively identified in 21 animals. Using bioinformatics, the authors concluded that gene coding errors likely prevent the successful production of GnRH2 in many species (21). A summary of the coding errors present in the *GnRH2* gene of mammals is available in Table 1. A premature stop codon truncates the mature decapeptide in the chimpanzee, orangutan, mouse lemur, sheep, and cat (21, 26), whereas the rabbit, pika, cow, dog, cat, and dolphin *GnRH2* genes encode an inactive peptide (21, 26). Early evidence implied that the rat and mouse also maintain a *GnRH2* gene as immunoreactive GnRH2 was detected in the rodent brain (27–29). Although it was later determined that the mouse genome only maintains a fragment of the *GnRH2* gene (exon 1) on chromosome 2 and it is completely deleted from chromosome 3 in the rat (21, 22, 30). Together, these data reveal that the *GnRH2* gene is absent or functionally inactivated in many mammals. In contrast, 10 species (human, macaque, marmoset, tarsier, tree shrew, guinea pig, musk shrew, common shrew, horse, and pig) maintain the appropriate genomic sequence to produce a biologically active decapeptide (21). We re-evaluated the presence of the *GnRH2* gene in mammals by surveying updated NCBI gene databases and found that an additional 68 mammals, comprising 9 additional orders, possess the *GnRH2* gene to date (Table 2). Thus, 89 mammals from 19 orders maintain the *GnRH2* gene, further suggesting that the *GnRH2* gene was present in a common mammalian ancestor (21). However, whether the *GnRH2* gene encodes a functional peptide in all of these animals remains unknown.

**Table 1:** Presence and potential functionality of the GnRH2 and GnRHR2 genes within mammals.^a^

Order	Mammal (genus species)	GnRH2			GnRHR2		
		Gene	Coding disruption^b^	Functional protein	Gene	Coding disruption^b^	Functional protein
*Artiodactyla*	Alpaca (*Vicugna pacos*)	+	?	?	+	?	?
	Cow (*Bos taurus*)	+	AAA	−	+	PSC	−
	Pig (*Sus scrofa*)	+	−	+	+	−	+
	Sheep (*Ovis aries*)	+	PSC	−	+	PSC	−

*Carnivora*	Cat (*Felis catus*)	+	PSC; FM; AAA	−	+	PSC	−
	Dog (*Canis familiaris*)	+	PSC; AAA	−	+	PSC; FM	−

*Cetacea*	Bottle-nosed dolphin (*Tursiops truncatus*)	+	AAA	−	+	FM; AAA; PSC	−

*Lagomorpha*	Pika (*Ochotona princeps*)	+	AAA; BI	−	?	?	?
	Rabbit (*Oryctolagus cuniculus*)	+	MS; PSC	−	+	MS; FM; PSC	−

*Perissodactyla*	Horse (*Equus caballus*)	+	−	+	+	FM; PSC	−

*Primates*	African green monkey (*Cercopithecus aethiops*)	?	?	?	+	−	+
	Chimpanzee (*Pan troglodytes*)	+	PSC	−	+	FM; PSC	−
	Common marmoset (*Callithrix jacchus*)	+	−	+	+	−	+
	Human (*Homo sapien*)	+	−	+	+	FM; PSC	−
	Mouse lemur (*Microcebus murinus*)	+	PSC	−	+	?	?
	Orangutan (*Pongo pygmaeus*)	+	PSC	−	+	−	+
	Rhesus macaque (*Macaca mulatta*)	+	−	+	+	−	+
	Tarsier (*Tarsius syrichta*)	+	−	+	+	?	?

*Proboscidea*	African elephant (*Loxodonta africana*)	+	?	?	+	−	+

*Rodentia*	Ground squirrel (*Spermophilus tridecemlineatus*)	+	?	?	+	PSC; AAA	−
	Guinea pig (*Cavia porcellus*)	+	−	+	+	PSC; FM	−
	Kangaroo rat (*Dipodomys ordii*)	+	?	?	+	AAA	?
	Mouse (*Mus musculus*)	+	GR	−	−	−	−
	Rat (*Rattus norvegicus*)	−	−	−	+	GR	−

*Scandentia*	Tree shrew (*Tupaia belangeri*)	+	−	+	+	−	+

*Soricomorpha*	Common shrew (*Sorex araneus*)	+	−	+	+	BD; FM; PSC	−
	Musk shrew (*Suncus murinus*)^c^	+	−	+	?	?	+

*^a^Data are based on bioinformatics. Absence of the gene or functional protein is denoted by a (−), whereas presence is indicated by a (+). Species in which there was not enough genomic information available (or the functionality has not yet been assessed) will be noted as a (?). Adapted from Stewart et al. (21)*.

*^b^In many species, the gene for *GnRH2* and/or *GnRHR2* is present but presumed non-functional due to gene coding errors. If the gene is affected, the nature of the disruption is indicated. Absence of a gene disruption is indicated by a minus symbol (−)*.

*^c^The genome of the musk shrew has not been fully annotated. Therefore, the presence of GnRHR2 in the musk shrew is putative based on the work of Temple et al. (31) and Kauffman et al. (32) establishing a functional GnRHR2*.

*Abbreviations: PSC, premature stop codon; FM, frameshift mutation; AAA, amino acid alteration; GR, gene remnant; BI, base insertion; BD, base deletion; MS, missing sequence*.

**Table 2:** Identification of the GnRH2 gene within 68 additional mammals *via* NCBI gene database queries.

Order	Common name	Genus species	NCBI gene ID
*Afrosoricida*	Cape golden mole	*Chrysochloris asiatica*	102818594

*Artiodactyla*	Alpaca	*Vicugna pacos*	102537645
	Arabian camel	*Camelus dromedarius*	105096036
	Bactrian camel	*Camelus bactrianus*	105083071
	Bison	*Bison bison bison*	104986175
	Goat	*Capra hircus*	102171992
	Texas white-tailed deer	*Odocoileus virginianus texanus*	110145856
	Tibetan antelope	*Pantholops hodgsonii*	102339298
	Water buffalo	*Bubalus bubalis*	102408995
	Wild bactrian camel	*Camelus ferus*	102511575
	Wild yak	*Bos mutus*	102280393
	Zebu cattle	*Bos indicus*	109567205

*Carnivora*	Amur tiger	*Panthera tigris altaica*	102950818
	Cheetah	*Acinonyx jubatus*	106980160
	Ferret	*Mustela putorius furo*	101672225
	Giant panda	*Ailuropoda melanoleuca*	100469838
	Leopard	*Panthera pardus*	109274781
	Pacific walrus	*Odobenus rosmarus divergens*	101378339
	Polar bear	*Ursus maritimus*	103674386
	Weddell seal	*Leptonychotes weddellii*	102734468

*Cetacea*	Killer whale	*Orcinus orca*	101276573
	Minke whale	*Balaenoptera acutorostrata scammoni*	103020677
	Sperm whale	*Physeter catodon*	102986533
	Yangtze river dolphin	*Lipotes vexillifer*	103085292

*Chiroptera*	Big brown bat	*Eptesicus fuscus*	103285836
	Brandt’s bat	*Myotis brandtii*	102246670
	Chinese rufous horseshoe bat	*Rhinolophus sinicus*	109448424
	Little brown bat	*Myotis lucifugus*	102433482
	Myotis davii bat	*Myotis davii*	102763285

*Dasyuromorphia*	Tasmanian devil	*Sarcophilus harrisii*	100916503

*Didelphimorphia*	Gray short-tailed opossum	*Monodelphis domestica*	103098126

*Diprotodontia*	Koala	*Phascolarctos cinereus*	110221117

*Erinaceomorpha*	Western European hedgehog	*Erinaceus europaeus*	103128681

*Eulipotyphla*	Star-nosed mole	*Condylura cristata*	101633945

*Macroscelidea*	Cape elephant shrew	*Elephantulus edwardii*	102862182

*Perissodactyla*	Donkey	*Equus asinus*	106844450
	Przewalski’s horse	*Equus przewalskii*	103555821
	Southern white rhinoceros	*Ceratotherium simum simum*	106802382

*Primate*	Angolan colobus	*Colobus angolensis*	105512752
	Black snub-nosed monkey	*Rhinopithecus bieti*	108529998
	Bolivian squirrel monkey	*Saimiri boliviensis*	101028145
	Coquerel’s sifaka monkey	*Propithecus coquereli*	105827546
	Crab-eating macaque	*Macaca fascicularis*	102124425
	Drill	*Mandrillus leucophaeus*	105535577
	Golden snub-nosed monkey	*Rhinopithecus roxellana*	104674617
	Green monkey	*Chlorocebus sabaeus*	103247081
	Ma’s night monkey	*Aotus nancymaae*	105711412
	Northern white-cheeked gibbon	*Nomascus leucogenys*	100594202
	Olive baboon	*Papio anubis*	100997952
	Pig-tailed macaque	*Macaca nemestrina*	105481609
	Pygmy chimpanzee	*Pan paniscus*	100971926
	Sooty mangabey monkey	*Cercocebus atys*	105582156
	Sumatran orangutan	*Pongo abelii*	100441160
	Sunda flying lemur	*Galeopterus variegatus*	103588840
	Western gorilla	*Gorilla gorilla*	101151325
	White-headed capuchin monkey	*Cebus capucinus imitator*	108286755
	White-tufted-ear marmoset	*Callithrix jacchus*	103792807

*Proboscidea*	African elephant	*Loxodonta africana*	100668639

*Rodentia*	Alpine marmot	*Marmota marmota marmota*	107143918
	American beaver	*Castor canadensis*	109686520
	Damara mole-rat	*Fukomys damarensis*	104853177
	Degu	*Octodon degus*	101565240
	Kangaroo rat	*Dipodomys ordii*	105981455
	Long-tailed chinchilla	*Chinchilla lanigera*	102005135
	Naked mole-rat	*Heterocephalus glaber*	101717034
	Thirteen-lined ground squirrel	*Spermophilus tridecemlineatus*	101971577

*Scandentia*	Chinese tree shrew	*Tupaia chinensis*	102500815

*Tubulidentata*	Aardvark	*Orycteropus afer afer*	103191804

### GnRH2 Is Ubiquitously Expressed within Mammals

Like GnRH1, GnRH2 has been identified in the pre-optic and medio-basal hypothalamic areas (7), albeit scarcely (33). Likewise, our group detected low levels of GnRH2 within the hypothalamus of the pig, implying that GnRH2 is not a prominent regulator of gonadotropin secretion (34, 35). GnRH2 has also been discovered in the midbrain and limbic structures, suggesting a role in the modulation of reproductive behavior (14, 29). In primates and humans, GnRH2 is prevalent in the caudate nucleus, hippocampus, and amygdala (36, 37) and has also been detected in the midbrain and hindbrain (7). White et al. (20) quantified expression of the *GnRH2* gene in 50 different human tissues, including numerous regions of the brain (20). Surprisingly, *GnRH2* mRNA was identified in all tissues examined and levels were highest in peripheral tissues; the converse was true for GnRH1.

In the periphery, transcript levels for *GnRH2* were 30-fold higher in the kidney and 4-fold higher in bone marrow and the prostate compared with the brain. GnRH2 is also produced in organs of the thoracic (e.g., heart, lung, and aorta), digestive (e.g., salivary gland, stomach, and intestine), endocrine (e.g., adrenal, pancreas, and thyroid), and immune (e.g., tonsil, leukocyte, and lymph node) systems (Table 3) (11, 29). Moreover, GnRH2 has been identified in numerous female (e.g., ovary, uterus, endometrium, and myometrium) and male (e.g., testis, epididymis, seminal vesicles, and prostate) reproductive organs (Table 3) (11, 29). The ubiquitous nature of this decapeptide is also evident in the many immortalized cell lines in which GnRH2 has been isolated, including cells derived from breast tissue (38), lymphocytes (39), ovaries (40, 41), and neural tissue (42). A summary of the mammalian cell lines that produce GnRH2 is available in Table 4. Ultimately, these data demonstrate that GnRH2 is ubiquitously expressed, indicating a divergent function from GnRH1.

**Table 3:** Production of GnRH2 and GnRHR2 in mammalian tissues.^a^

Tissue or cell type	GnRH2		GnRHR2		Reference
	Identified^b^	Species	Identified^b^	Species	
**Central nervous system**					
Brain (whole)	+	h	+	h	(11, 20, 29)
Forebrain^c^	+	h, r	+	h, r, m	(16, 20, 29)
Midbrain^c^	+	h, r	+	h, r, m	(16, 20, 29)
Hindbrain^c^	+	r	+	h, r, m	(16, 20, 29)
Spinal cord	+	h	+	h, m	(20, 29)

**Endocrine**					
Hypothalamus	+	p	+	m	(29, 35)
Pituitary (whole)	+	h	+	m, h	(11, 20, 29)
Anterior pituitary	+	p	+	p	(35)
Adrenal gland	+	h	+	h, m	(11, 20, 29)
Pancreas	+	h	+	h, m	(16, 20, 29)
Thyroid	+	h	+	h, m	(11, 20, 29)

**Thoracic**					
Heart	+	h	+	h, m	(11, 16, 20, 29)
Aorta	+	h			(20)
Lung	+	h	+	h, m	(11, 20, 29)
Thymus gland	+	h	+	h, m	(11, 20, 29)
Trachea	+	h			(20)

**Digestion and metabolism**					
Salivary gland	+	h			(20)
Stomach	+	h	+	h, m	(11, 20, 29)
Small intestine	+	h	+	h	(11, 20)
Duodenum	−	h			(43)
Jejunum	+	h			(43)
Ileum	+	h			(43)
Large intestine	+	h	+	h	(11, 20)
Cecum	+	h			(43)
Colon	−	h			(43)
Rectum	−	h			(43)
Liver	+	h	+	h, m	(11, 16, 20, 29)
Skeletal muscle	+	h	+	m, h	(11, 16, 20, 29)

**Renal**					
Bladder	+	h	+	m	(20, 29)
Kidney	+	h	+	h, m	(11, 16, 20, 29)

**Immune**					
Peripheral leukocyte	+	h			(20)
T lymphocyte	+	h			(39)
Lymph node	+	h			(20)
Tonsil	+	h			(20)
Bone marrow	+	h			(20)
Spleen	+	h	+	h, m	(11, 20, 29)

**Female reproductive**					
Ovary	+	h	+	h, m	(11, 20, 29)
Ovarian surface epithelial cells	+	h			(40, 41)
Granulosa cells	+	h			(41)
Oviduct			+	m	(29)
Uterus	+	h	+	h, m	(11, 20, 29)
Endometrium	+	h			(20)
Myometrium	+	h	+	h	(44)
Breast/mammary	+	h	+	h, m	(11, 20, 29, 38)
Placenta	+	h	+	h	(11, 20)

**Male reproductive**					
Testis	+	h, p	+	h, m, p	(11, 20, 29, 35, 45)
Leydig cells	+	p	+	p	(35, 45)
Sertoli cells	+	p	+	p	(35, 45)
Germ cells	+	p	+	h, p	(35, 45, 46)
Spermatozoa			+	h, p	(34, 45, 46)
Epididymis	+	p	+	m, p	(29, 34)
Seminal vesicles	+	p	+	m, p	(29, 34)
Bulbourethral	+	p	+	p	(34)
Prostate	+	h, p	+	h, m, p	(11, 20, 29, 34)

*^a^Adapted from Millar (16). Blank cells indicate tissues that have not yet been examined*.

*^b^Either mRNA or protein was discovered*.

*^c^See Millar (16) for specific regions of the brain that produce GnRH2 and/or GnRHR2*.

*Abbreviations: +, positive; −, negative; p, pig; h, human; m, marmoset; r, rhesus macaque*.

**Table 4:** Production of GnRH2 and GnRHR2 in mammalian cell lines.

Cell line	Origin	Species	GnRH2^a^	GnRHR2^a^	Reference
**Nervous**					
TE671	Neuronal medulloblastoma	Human	+		(41)

**Lung**					
A549	Alveolar adenocarcinoma	Human		+	(11)

**Digestive**					
SW480	Colorectal adenocarcinoma	Human		+	(11)
IPEC-J2	Intestinal epithelial cells	Porcine		+	(48)

**Immune**					
HL-60	Promyelocytic leukemia	Human		+	(11)
Jukat	T cell leukemia	Human	+	+	(39, 49)

**Mammary**					
MDAMB-231	Breast adenocarcinoma	Human	+		(38)
MCF-7	Breast adenocarcinoma	Human	+		(38)
MCF-10A	Breast epithelium	Human	+		(38)

**Female reproductive**					
HeLa	Cervical adenocarcinoma	Human		+	(11, 50)
Hec-1A	Endometrial adenocarcinoma	Human		+	(51)
Hec-1B	Endometrial adenocarcinoma	Human			(52)
Ishikawa	Endometrial adenocarcinoma	Human		+	(51)
HHUA	Endometrial adenocarcinoma	Human		+	(49)
EFO-21	Ovarian cystadenocarcinoma	Human		+	(51)
EFO-27	Ovarian adenocarcinoma	Human		−	(52)
OVCAR-3	Ovarian adenocarcinoma	Human	+	+	(40, 51)
SK-OV-3	Ovarian adenocarcinoma	Human	+	+	(40, 51)
CaOV-3	Ovarian adenocarcinoma	Human	+		(40)
BG-1	Ovarian adenocarcinoma	Human	+	+	(47, 52)
IOSE-29	Ovarian surface epithelia	Human	+		(40)
SVOG-4O	Granulosa-luteal cells	Human	+		(41)
SVOG-4m	Granulosa-luteal cells	Human	+		(41)

**Male reproductive**					
ST	Fetal testis	Porcine		+	(53)
ALVA-41	Prostate adenocarcinoma	Human		+	(50)
PPC-1	Prostate adenocarcinoma	Human		+	(50)
DU-145	Prostate carcinoma	Human		+	(49, 50)

**Urinary**					
TSU-Pr1	Bladder carcinoma	Human		+	(49, 50)
COS-1^b^	Kidney	African green monkey		+	(11)
HEK293	Embryonic kidney	Human		+	(50)

*^a^The presence of a (+) indicates that either mRNA or protein has been identified, whereas a (−) specifies that the tissue was negative. Blanks designate cell lines that have not yet been examined*.

*^b^The presence of GnRHR2 protein is putative as GnRH2 treatment of COS-1 cells yielded IP accumulation*.

### Transcriptional Regulation of GnRH2 Gene Expression

The expression of *GnRH2* is regulated by several different reproductive hormones including androgens, 17β-estradiol, progesterone, the gonadotropins, and GnRH2 itself. Darby et al. (54) demonstrated in tumors from prostate cancer patients that androgens enhance *GnRH2* expression. This effect was likely mediated at the transcriptional level because sequence analysis of the 5′ flanking region for the human *GnRH2* gene revealed the presence of a putative androgen response element and direct interaction with the androgen receptor was confirmed *via* chromatin immunoprecipitation assays (54). In females, *GnRH2* expression in the hypothalamus of rhesus macaques is stimulated by 17β-estradiol treatment (55). Similarly, treatment of human granulosa-luteal cells with 17β-estradiol resulted in a dose-dependent increase in *GnRH2* mRNA expression (56) and 17β-estradiol exerted a stimulatory effect on *GnRH2* expression in human neuronal cells (42). The effect of progesterone has also been examined in humans. Primary cultures of human granulosa-luteal cells treated with RU486 (progesterone receptor antagonist) increased *GnRH2* expression in a time- and dose-dependent fashion (56). However, neither progesterone nor RU486 affected *GnRH2* expression in human neuronal cells (57).

There is also evidence that protein hormones modulate GnRH2 production. Treatment with FSH or human chorionic gonadotropin (hCG) upregulated *GnRH2* gene transcription in granulosa-luteal cells of humans (58). Likewise, *GnRH2* mRNA and protein levels increased in human neuronal cells in response to cAMP treatment, a downstream messenger of the gonadotropins (59). This effect probably occurs at the transcriptional level given that mutation of a putative cAMP-responsive element in the 5′ flanking sequence of the human *GnRH2* gene suppressed activity of the *GnRH2* promoter (59). However, when normal and cancerous ovarian cells were treated with LH and FSH, *GnRH2* expression was reduced in the majority of cell lines tested; only CaOV-3 and SKOV-3 cells were unaffected by treatment, despite expression of receptors for the gonadotropins (47). GnRH2 may mediate its own expression in an autocrine/paracrine manner. For example, granulosa cells secrete GnRH2 (60) and culture of human granulosa-luteal cells for 10 days increased *GnRH2* mRNA expression (56). A different study demonstrated that treatment of luteinized granulosa cells with GnRH2 downregulated *GnRH2* expression (58).

### Prepro-GnRH2

The porcine prepro-GnRH2 is only 56% homologous to prepro-GnRH1 (NCBI accession numbers XP_005672842 and NP_999439, respectively) but contains the same components: a signal sequence, the decapeptide, a conserved cleavage site, and GAP (20). As with all peptide hormones, the signal sequence directs the hormone to the secretory pathway (61). Interestingly, the signal sequence of prepro-GnRH1 and prepro-GnRH2 are dissimilar in the pig (Figure 1C). This distinction could be important because composition of the signal sequence has been reported to influence the efficiency of secretion (61). The cleavage site (Gly–Lys–Arg) is conserved between prepro-GnRH2 and prepro-GnRH1 (Figure 1C), indicating that carboxypeptidase E, the enzyme responsible for cleaving GnRH1 from GAP in mice (62), likely processes prepro-GnRH2 as well.

To date, the GAP of prepro-GnRH2 has not been studied directly. However, the GAP of prepro-GnRH1 is secreted with the mature decapeptide (63) and has been associated with prolactin and gonadotropin secretion (64, 65). Prepro-GnRH1 and prepro-GnRH2 in the human have similar lengths except the GAP, which is 50% longer in prepro-GnRH2 (20). Porcine prepro-GnRH2 (110 amino acids) is also longer than prepro-GnRH1 (91 amino acids), primarily due to a longer GAP (73 versus 55 amino acids, respectively; Figure 1C). A similar result was also reported for the tree shrew (66), indicating that a longer GAP in prepro-GnRH2 may be common in mammals (20) and could have functional relevance.

Notably, White et al. (20) reported the presence of two GnRH2-GAP variants in humans. Certain tissues (e.g., fetal brain and thalamus) expressed a longer GAP variant than others (e.g., kidney) (20). Likewise, Cheon et al. (67) discovered two transcript variants of *GnRH2* with differing GAP lengths in the endometrium of women. The porcine *GnRH2* gene is also predicted to produce two forms of prepro-GnRH2. The classical product is 110 amino acids (NCBI accession number XP_005672842), whereas the splice variant encodes a 143 amino acid isoform (NCBI accession number XP_013840618), due to the retention of intron 2. The only differences between the two products were detected in the GAP region (Figure 1C). Although, the biological significance of these variants of GAP for prepro-GnRH2 has not yet been elucidated.

### The Structure of GnRH2

GnRH2 (pGlu–His–Trp–Ser–His–Gly–Trp–Tyr–Pro–Gly–NH_2_) differs from GnRH1 by three amino acids [His^5^, Trp^7^, and Tyr^8^; (12)], resulting in 70% sequence identity (20). Amino acids His^5^, Trp^7^, and Tyr^8^ help stabilize GnRH2, whereas the N-terminal (pGlu^1^, His^2^, Trp^3^, and Ser^4^) and C-terminal (Pro^9^, Gly–NH_2_^10^) residues are essential for receptor binding and activation (68). Structurally, GnRH2 is more negatively charged and slightly bulkier than GnRH1 (30). GnRH2 has a β-turn conformation that is similar to GnRH1; however, GnRH2 exists in a preconfigured conformation. Thus, GnRH2 does not require extensive conformational changes for receptor activation (68). The conformation of GnRH2 may render it less sensitive to peptidases (69), likely increasing (6-fold) its stability (70) and half-life (71, 72) compared with GnRH1, which is rapidly degraded (73).

## Gonadotropin-Releasing Hormone 2 Receptor

### Identification of GnRHR2 in Mammals

Originally cloned in African catfish (74), a 7-transmembrane (TM) G protein-coupled receptor (GPCR) specific to GnRH2 (GnRHR2) has also been discovered in mammals (11, 29). The *GnRHR2* gene was first identified in mammals by surveying the human genome for genes with high homology to *GnRHR1* (11, 29). A gene with 40% homology to *GnRHR1* was identified but it was more similar to *GnRHR2* in fish (65% identity) and amphibians (55% identity), suggesting the identification of a human *GnRHR2* gene (11). Mammals only maintain genes for *GnRHR1* and *GnRHR2*, although a *GnRHR3* gene has been discovered in other vertebrates (16).

### The Gene for GnRHR2

**Figure 2:**
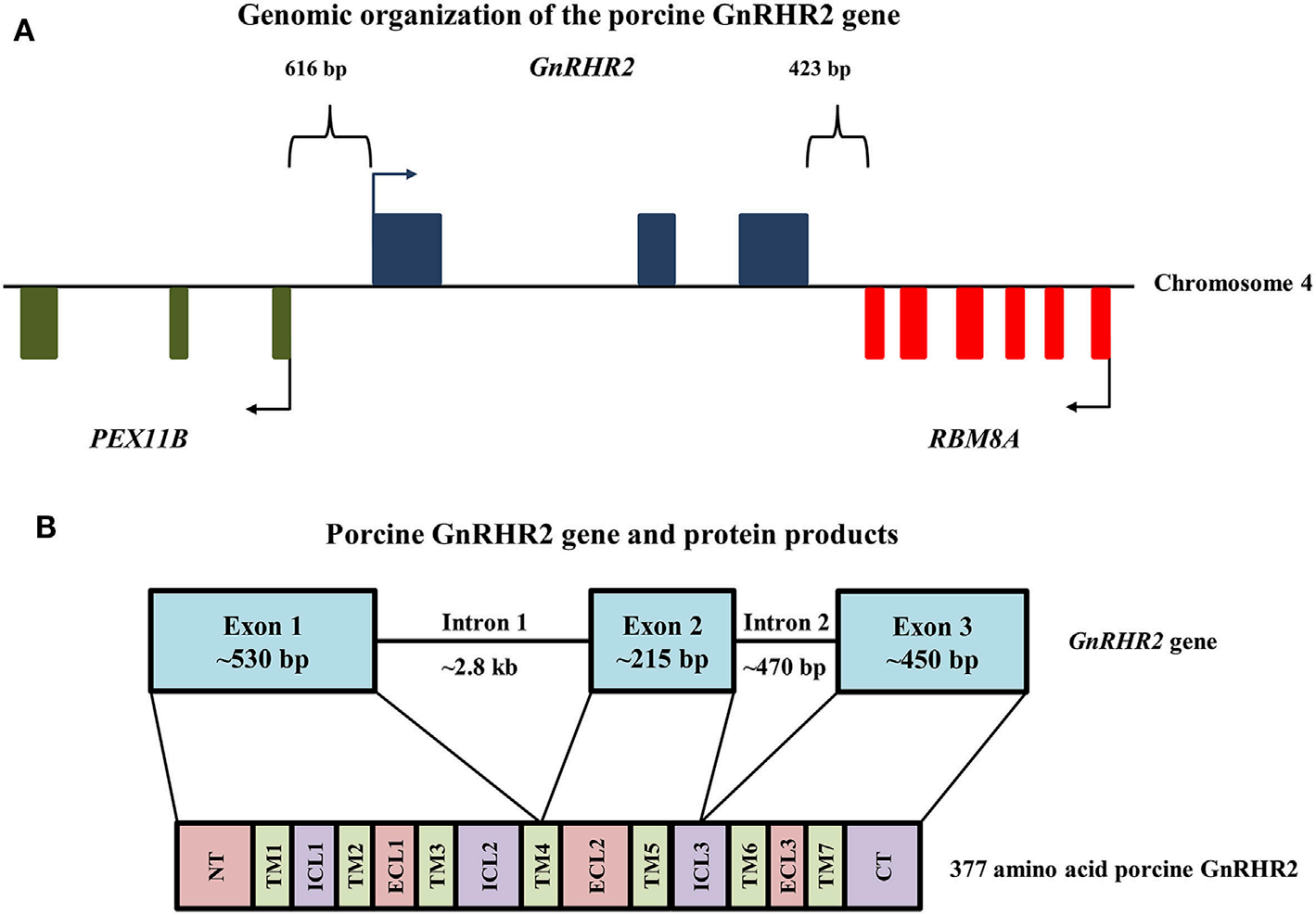
Genomic organization of the porcine GnRHR2 gene on chromosome 4. **(A)** The *PEX11B* gene (green) is located 616 bp upstream, whereas the *RBM8A* gene (red) is positioned 423 bp downstream of the *GnRHR2* gene (blue). Arrows indicate start codons for each gene. Reprinted from Brauer et al. (75) with permission from Elsevier. **(B)** The *GnRHR2* gene contains 3 coding exons and 2 introns, yielding a 377 amino acid product. Exon 1 of the porcine *GnRHR2* gene encodes the extracellular N-terminus (NT), transmembrane (TM) domains 1–3, part of TM4, intracellular loops (ICL) 1–2 and extracellular loop (ECL) 1. Exon 2 encodes the remainder of TM4 as well as TM5, ECL2, and part of ICL3. Exon 3 encodes the remainder of ICL3, TM domains 6–7, ECL3, and the C-terminal tail (CT). Note that introns and exons are not drawn to scale.

The human, chimpanzee, and rhesus macaque *GnRHR2* gene is located on chromosome 1 (21). In other species, however, the gene for *GnRHR2* is located on chromosome 17 (dog), chromosome 5 (horse), chromosome 3 [cow; (21)], and chromosome 4 [pig; (21, 75)]. The *GnRHR2* gene is closely associated in the antisense orientation with the *RBM8A* and *PEX11B* genes in all species (21, 22, 76). In humans, the promoter regions of *GnRHR2* and *PEX11B* overlap and the 3′ untranslated region (UTR) of *RBM8A* overlaps with two exons of the *GnRHR2* gene (76, 77). The *RBM8A* gene encodes RNA-binding motif protein 8A, which helps modulate the post-translational regulation of gene expression (78) and the *PEX11B* gene codes for peroxisomal membrane protein 11β, which is involved in the regulation of peroxisome abundance (79). In the pig, the *RBM8A* gene is located 423 bp downstream of the *GnRHR2* gene (Figure 2A). The 3′ UTRs of these two genes overlap, in opposite orientation on complementary strands. Upstream of the *GnRHR2* gene, the *PEX11B* gene is also located on the antisense strand in the opposite orientation (Figure 2A). The proximal promoters of these two genes overlap, with start codons 616 bp apart (75).

The porcine *GnRHR2* gene has three exons and two introns (Figure 2B). Exon 1 (530 bp) of the porcine *GnRHR2* gene encodes the extracellular N-terminus, TM domains 1–3, part of TM domain 4, intracellular loop (ICL) 2, and extracellular loop (ECL) 1. Exon 2 (215 bp) encodes the remainder of TM4 as well as TM5, ECL2, and part of ICL3. Exon 3 (450 bp) encodes the remainder of ICL3, TM6–7, ECL3, and the C-terminal tail (Figure 2B). Intron 1 of the porcine *GnRHR2* gene is relatively large (2.8 kb) compared with intron 2 (470 bp; Figure 2B), similar to reports from other species (21).

An additional truncated *GnRHR2* gene has also been discovered on chromosome 14 in humans (80), containing only exons 2 and 3 (46). Interestingly, exons 2 and 3 of this apparent pseudogene are 100% identical to the corresponding exons of the complete *GnRHR2* gene on chromosome 1 (80). This distinction may be especially critical when designing primers/probes for analysis of *GnRHR2* gene expression in human tissues. The truncated *GnRHR2* gene (chromosome 14) is more transcriptionally active and widely expressed than the full-length gene [chromosome 1; (46)]. This could explain discrepancies in *GnRHR2* mRNA levels of human tissues across studies performed prior to this finding; Neill et al. (11) utilized a riboprobe specific to exon 3, whereas Millar et al. (29) used a DNA probe specific to exon 1 (46). Consistent with the full-length gene on chromosome 1, this truncated *GnRHR2* gene is flanked by *RBM8A*, indicating it was duplicated from the chromosome 1 locus *via* retrotransposition (77). In fact, transcripts of this truncated gene contain exon 2 and 3, retain intron 2, and also include the 3′ UTR of *RBM8A*, except in the antisense orientation (46). Likewise, the genome of the African elephant contains a *GnRHR2* pseudogene with several point mutations/deletions and lacking exon 1 (21).

### Presence of the GnRHR2 Gene in Mammals

Using bioinformatics, the *GnRHR2* gene was identified in the genomes of 22 mammalian species, although sequence analysis revealed that gene disruptions occurred in 12 of these species [Table 1; (21)]. Early evidence implied that the *GnRHR2* gene was functional in the human, sheep, and mouse as immunostaining for GnRHR2 was detected in brain tissue of these species (29). However, the ovine *GnRHR2* gene contains a premature stop codon in exon 1 and a 51 bp deletion in exon 2, preventing translation of a full-length protein (26, 81). In humans (and chimpanzees), a frameshift mutation occurs due to a missing nucleotide (compared with the marmoset) in the 5′ flanking sequence. A premature stop codon is also present due to a single base change in an arginine codon in exon 2 (26, 81). Furthermore, the *GnRHR2* gene was subsequently determined to be absent from chromosome 3 in the mouse genome (21, 22) and only a remnant of exon 1 remains on chromosome 2 in the rat (22, 30). In other species, the *GnRHR2* gene likely encodes a non-functional protein as well. The bovine *GnRHR2* gene is disrupted by frameshift mutations in all three exons, in addition to premature stop codons in exons 2 and 3 (26). The squirrel *GnRHR2* gene contains a premature stop codon and an amino acid substitution, whereas the guinea pig has frameshift mutations in all three exons and two premature stop codons in exon 3 (21). The rabbit gene has a 14 bp deletion that results in a frame shift and premature stop codon, and the cat gene has a premature stop codon (21). In addition, the *GnRHR2* genes in the common shrew, dolphin, horse, and dog harbor several frameshifts and premature stop codons (21). In fact, only eight species (orangutan, African green monkey, rhesus macaque, marmoset, tree shrew, kangaroo rat, pig, and elephant) possess the appropriate gene sequence to produce a functional GnRHR2 [Table 1; (21)].

Our laboratory surveyed the updated NCBI gene databases to re-evaluate which mammals maintain the *GnRHR2* gene. The *GnRHR2* gene was confirmed in the genomes of 61 additional species representing 11 more orders (Table 5). Based on this report and Stewart et al. (21), the gene for *GnRHR2* is present in the genomes of 83 species to date, encompassing 22 different mammalian orders. However, it is unclear if the gene is functional or silenced in these animals. Therefore, future bioinformatics work is needed to examine the characteristics of the *GnRHR2* gene within these species.

**Table 5:** Identification of the GnRHR2 gene within 61 additional mammals *via* NCBI gene database queries.

Order	Common name	Genus species	NCBI gene ID
*Afrosoricida*	Cape golden mole	*Chrysochloris asiatica*	102817241
	Lesser hedgehog tenrec	*Echinops telfairi*	101659402

*Artiodactyla*	Alpaca	*Vicugna pacos*	102526131
	Arabian camel	*Camelus dromedaries*	10510588
	Bactrian camel	*Camelus bactrianus*	105079482
	Bison	*Bison bison bison*	104983739
	Goat	*Capra hircus*	102184212
	Texas white-tailed deer	*Odocoileus virginianus texanus*	110144360
	Wild bactrian camel	*Camelus ferus*	102518691
	Wild yak	*Bos mutus*	102266467
	Zebu cattle	*Bos indicus*	109555832

*Carnivora*	Amur tiger	*Panthera tigris altaica*	102969203
	European domestic ferret	*Mustela putorius furo*	101687337
	Giant panda	*Ailuropoda melanoleuca*	100484454
	Pacific walrus	*Odobenus rosmarus*	101379215
	Polar bear	*Ursus maritimus*	103678407
	Weddell seal	*Leptonychotes weddellii*	102742574

*Cetacea*	Killer whale	*Orcinus orca*	101271242
	Minke whale	*Balaenoptera acutorostrata scammoni*	103006588
	Sperm whale	*Physeter catodon*	102982977
	Yangtze river dolphin	*Lipotes vexillifer*	103074337

*Cingulata*	Nine-banded armadillo	*Dasypus novemcinctus*	101438616

*Dasyuromorphia*	Tasmanian devil	*Sarcophilus harrisii*	100925975

*Didelphimorphia*	Gray short-tailed opossum	*Monodelphis domestica*	100014969

*Diprotodontia*	Koala	*Phascolarctos cinereus*	110219988

*Erinaceomorpha*	Western European hedgehog	*Erinaceus europaeus*	103127802

*Eulipotyphla*	Star-nosed mole	*Condylura cristata*	101623053

*Macroscelidea*	Cape elephant shrew	*Elephantulus edwardii*	102866982

*Perissodactyla*	Donkey	*Equus asinus*	106847655
	Przewalski’s horse	*Equus przewalskii*	103556230
	Southern white rhinoceros	*Ceratotherium simum simum*	101400748

*Pholidota*	Malayan pangolin	*Manis javanica*	108400376

*Primates*	Angolan colobus	*Colobus angolensis*	105518331
	Black snub-nosed monkey	*Rhinopithecus bieti*	108531432
	Bolivian squirrel monkey	*Saimiri boliviensis*	101045352
	Coquerel’s sifaka monkey	*Propithecus coquereli*	105809593
	Crab-eating macaque	*Macaca fascicularis*	102142398
	Drill	*Mandrillus leucophaeus*	105550280
	Golden snub-nosed monkey	*Rhinopithecus roxellana*	104671044
	Green monkey	*Chlorocebus sabaeus*	103225877
	Ma’s night monkey	*Aotus nancymaae*	105730634
	Mouse lemur	*Microcebus murinus*	109730387
	Northern white-cheeked gibbon	*Nomascus leucogenys*	100593225
	Olive baboon	*Papio anubis*	101008793
	Pig-tailed macaque	*Macaca nemestrina*	105479042
	Pygmy chimpanzee	*Pan paniscus*	100995783
	Small-eared galago	*Otolemur garnettii*	100963950
	Sooty mangabey monkey	*Cercocebus atys*	105592861
	Sumatran orangutan	*Pongo abelii*	100460080
	Sunda flying lemur	*Galeopterus variegatus*	103595864
	Tarsier	*Tarsius syrichta*	103268450
	Western gorilla	*Gorilla gorilla*	101137503
	White-headed capuchin monkey	*Cebus capucinus imitator*	108293668
	White-tufted-ear marmoset	*Callithrix jacchus*	100399755

*Rodentia*	Alpine marmot	*Marmota marmota marmot*	107160395
	American beaver	*Castor canadensis*	109682308
	Damaraland mole rat	*Fukomys damarensis*	104865794
	Degu	*Octodon degus*	101580342
	Naked mole-rat	*Heterocephalus glaber*	101703276

*Scandentia*	Chinese tree shrew	*Tupaia chinensis*	102498810

*Sirenia*	Florida manatee	*Trichechus manatus latirostris*	101357387

*Tubulidentata*	Aardvark	*Orycteropus afer afer*	103203244

Coupled with the aforementioned *GnRH2* gene distribution data, it is clear that few mammalian species have a functional GnRH2–GnRHR2 system. The maintenance of only part of the system in some species (e.g., ligand or receptor only) assumes interaction with the GnRH1–GnRHR1 system to maintain functionality. Millar et al. (18) proposed that the presence of GnRH2, but not a functional GnRHR2, in some species signifies that GnRHR1 has adopted a dual role for the actions of GnRH1 and GnRH2 through alternative ligand conformations and downstream signaling events. In species that produce both GnRH2 and its receptor, however, this system was likely critical to survival to have avoided gene inactivation throughout evolution.

### Does the Human Produce the GnRHR2?

When GnRHR2 was first discovered in mammals, GnRHR2-specific immunostaining was detected in the human brain (29), implying that humans produce a full-length GnRHR2. However, it was later discovered that coding errors likely interrupt successful translation of human *GnRHR2* mRNA (21). Indeed, Neill et al. (82) reported the inability to identify translatable *GnRHR2* transcripts that would yield a full-length receptor in humans, at least *via* conventional mechanisms. Recall, the human *GnRHR2* gene contains a frameshift mutation in exon 1 and a premature stop codon in exon 2 (76, 82). Yet, the gene remains transcriptionally active and produces transcript variants due to alternative splicing, suggesting functionality as most pseudogenes are promoterless (76). These conflicting results have been the subject of much research and debate.

Despite the apparent gene coding errors, there is evidence for functionality of the GnRHR2 in humans (82). For example, use of a GnRHR1 antagonist mitigated the effects of GnRH1, but not GnRH2, in human decidual stromal (83) and trophoblast (84) cells. GnRH2, and not GnRH1, effectively suppressed proliferation of SK-OV-3 cells containing only *GnRHR2* mRNA (51). In human cancer cells with reduced GnRHR1 levels, GnRH2 (not GnRH1) retained the ability to inhibit cell proliferation (52). Small interfering RNA targeting *GnRHR1* inhibited the actions of GnRH1 on trophoblast invasion, but GnRH2-mediated effects persisted (84). GnRH1 and GnRH2 also have differing effects in primary cultures of human decidual stromal cells; GnRH1 increased whereas GnRH2 suppressed mRNA and protein levels (83). Although the results of the latter study could be related to divergent signaling of GnRH2 at the GnRHR1, these data ultimately support the presence of a functional GnRHR2 in humans.

Many hypotheses have arisen for how the disrupted *GnRHR2* gene may retain functionality including: (1) counteractive shifts in the reading frame, (2) recoding of stop codons, (3) alternative splicing, (4) alternative protein translation, or (5) production and functionality of GnRHR2 fragments (82). The mechanisms that could potentially yield a functional receptor from a seemingly non-translatable mRNA sequence were reviewed by Neill et al. (82). First, a corrective shift in the reading frame, albeit rare, has been demonstrated in eukaryotes, allowing for the production of a full-length protein (85). Indeed, Millar et al. (18) isolated human *GnRHR2* transcripts missing the stop codon. Second, the use of an alternative start codon (e.g., GUG instead of AUG) has also been considered. In humans, a GUG codon is downstream of the frameshift and would yield a 5-TM receptor with a truncated (22 amino acids) N-terminus. Interestingly, a similar phenomenon occurs in the translation of African green monkey *GnRHR2* mRNA (11, 21, 82). Furthermore, the GUG codon within the human transcript meets the two Kozak criteria necessary for an alternative start codon (86, 87). Third, it has been proposed that the premature stop codon may be recoded (82), which occurs in mammals (85, 88, 89). In the human *GnRHR2* mRNA transcript, the premature stop codon is UGA but this codon can also encode for the amino acid, selenocysteine, potentially preventing the termination of translation (16, 46, 76, 80). However, attempts to identify selenocysteine incorporation thus far have been fruitless (76). Moreover, alternative splicing in exon 1 would yield a 5-TM *GnRHR2* transcript without the reading frame disruption near the N-terminus of the 7-TM isoform (82). The premature stop codon, however, would still be present. Therefore, translation of 5-TM receptor mRNA would also require a stop codon read-through. A fourth hypothesis is that fragments of both the 5- and 7-TM isoforms are produced and reassociate non-covalently after translation, a characteristic that has been shown with other GPCRs, including GnRHR1 (90–92). Data from Grosse et al. (92) showed that two coexpressed GnRHR1 fragments (corresponding to TM domains 1–5 and 6–7) reassociated to produce a full-length, functional receptor. If protein fragments of the 7-TM GnRHR2 isoform (containing TM domains 1–4 and 6–7) reassociated, they would form a 6-TM isoform. However, it is unclear if a 6-TM receptor would be functional (82).

The final theory is that GnRHR2 fragments are successfully produced and modulate GnRHR1 activity (82). This hypothesis is seemingly supported by the work of Pawson et al. (93) who demonstrated that a GnRHR2 protein fragment (termed the GnRHR2 reliquium) inhibits post-translational GnRHR1 protein abundance *via* interactions with GnRHR1 inside the nucleus, endoplasmic reticulum, and/or Golgi apparatus (93). This fragment spans the cytoplasmic end of the 5-TM domain to the carboxyl terminus of the full-length receptor and would be produced by the aforementioned alternative start site (GUG) downstream of the premature stop codon (93). Interestingly, others have reported that GnRHR1 fragments with similar domains (TM6–7) suppress GnRH-mediated signaling when coexpressed with the full-length GnRHR1 (92). This theory is supported by early work revealing GnRHR2 immunostaining in human tissues with an antibody (ZGRH-II-5) directed against ECL3 (29), which is retained in the GnRHR2 reliquium (93). Likewise, the ovine *GnRHR2* gene may produce a GnRHR2 reliquium as well, given that ZGRH-II-5 antiserum also revealed GnRHR2 immunostaining in the sheep brain (93). In contrast, a different antibody directed against ECL2 (absent in the GnRHR2 reliquium) failed to indicate GnRHR2 immunostaining in sheep (93). Notably, mRNA that encodes for the GnRHR2 reliquium has been successfully identified in many human organs and cell lines, suggesting that this protein fragment may be physiologically relevant (76). In addition, when the full-length cDNA sequence for human *GnRHR2* (including frame shift and premature stop codon) is transiently transfected into COS cells, expression of *GnRHR1* is enhanced (18). Thus, it is plausible that a functional (albeit unconventional) GnRHR2 is produced in humans. To date, however, the controversy remains unresolved and actions of GnRH2 in humans are predominantly ascribed to GnRHR1 signaling.

### Characterization of the Mammalian GnRHR2 Gene Promoter

**Figure 3:**
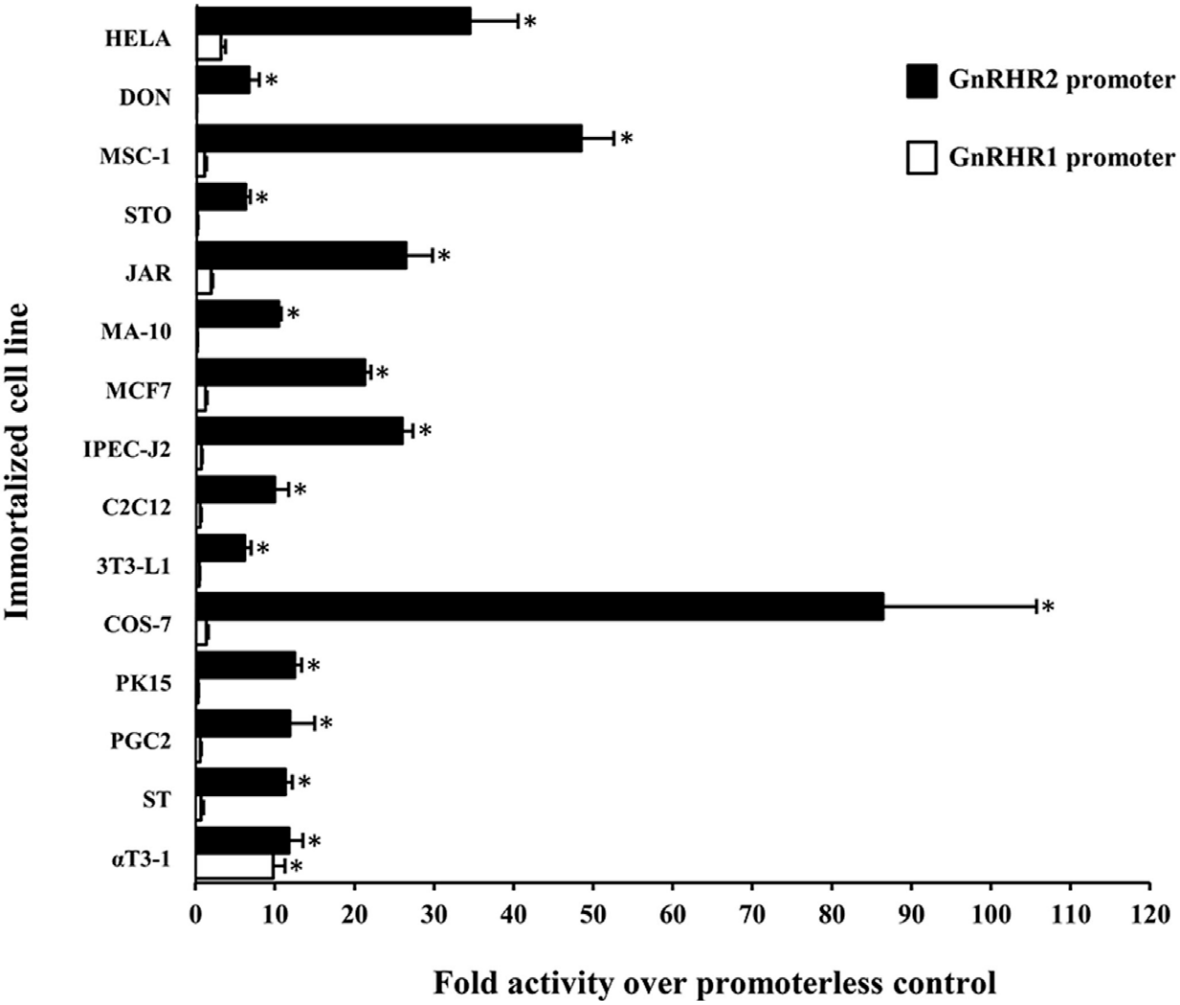
Activity of the porcine GnRHR1 and GnRHR2 gene promoters within different mammalian cell lines. Cells were transiently transfected with either the porcine *GnRHR1* (white) or *GnRHR2* (black) gene promoter in a luciferase reporter vector and expressed as fold activity over the promoterless control. Abbreviations: HELA, human cervical cancer; DON, Chinese hamster lung; MSC-1, mouse Sertoli cell; STO, mouse embryonic fibroblast; JAR, human placenta; MA-10, mouse Leydig cell; MCF7, human breast cancer; IPEC-J2, piglet intestinal epithelium; C2C12, mouse muscle myoblast; 3T3-L1, mouse adipocyte fibroblast; COS-7, green monkey kidney; PK15, porcine kidney; PGC-2, porcine granulosa cell; ST, swine testis; and αT3-1, murine gonadotrope. An asterisk indicates Least Squares Means that are significantly greater than the promoterless control (*P* < 0.05). Reprinted from Brauer et al. (75) with permission from Elsevier.

While transcriptional regulation of the *GnRHR1* gene has been evaluated in mice (94), rats (95), humans (96), sheep (97), and pigs (98), much less is known regarding the regulation of *GnRHR2* gene expression. The *GnRHR2* gene is transcriptionally active in several mammals, including humans, sheep, monkeys, marmosets, musk shrews, and pigs (11, 32, 46, 81, 82, 99). However, the regulatory elements governing the expression of this gene have only been studied in the marmoset and pig (75, 99). Utilizing luciferase reporter constructs containing either the *GnRHR1* or *GnRHR2* pig promoter in transient transfection assays with cell lines from several tissues, the *GnRHR2* promoter was active in all cell types examined, whereas activity of the *GnRHR1* promoter only exceeded the promoterless control in gonadotrope-derived αT3-1 cells [Figure 3; (75)]. Initial studies in immortalized swine testis (ST) cells revealed that activity of the porcine *GnRHR2* promoter was partially conferred by nuclear factor-κB, specificity protein 1 and 3 (SP1/3), and overlapping early growth response 1/SP1/3 (EGR1/SP1/3)-binding sites (75). The EGR1 and SP1/3-binding sites are located in a region of the 5′ UTR that is highly conserved compared with the marmoset *GnRHR2* promoter and previously shown to enhance promoter activity (99). Given the ubiquitous expression of *GnRHR2*, it was not surprising that a transcription factor such as SP1, also considered to be widely produced, would be involved in regulation. This contrasts greatly from the three steroidogenic factor 1-binding elements required for basal expression of the porcine *GnRHR1* gene in the gonadotrope-derived αT3-1 cell line (98). So, in accord with divergent expression patterns of the *GnRHR1* and *GnRHR2* genes, their transcription is differentially regulated as well.

### The GnRHR2 Gene Is Ubiquitously Expressed in Mammals

Similar to GnRH2, the *GnRHR2* gene is widely expressed throughout the body (11, 29). Within the brain, the GnRHR2 was found in the forebrain, midbrain, and hindbrain (16). Expression was pronounced in areas that regulate sexual behavior, such as the putamen, occipital lobe, cerebellum, and caudate nucleus, but reduced within the anterior pituitary gland (29). In addition, *GnRHR2* mRNA was also found in peripheral tissues including the heart, stomach, intestine, kidney, spleen, skeletal muscle, thymus, lung, liver, pancreas, adrenal, thyroid, placenta, uterus, ovary, breast, seminal vesicles, epididymis, prostate, and testis [Table 3; (11, 29)]. Relative to *GnRHR2* mRNA amounts in the pituitary, expression levels were lowest in the marmoset bladder and highest in the testis (29). Likewise, van Biljon et al. (46) detected strong *GnRHR2* signal in the human testis *via in situ* hybridization and our laboratory reported abundant GnRHR2 protein levels in the testis compared with the anterior pituitary gland of the pig (35). The receptor has been found in reproductive cancer cell lines derived from the prostate, cervix, endometrium and ovary, as well as cell lines produced from other organ systems (e.g., respiratory, digestive, mammary, immune, and urinary; Table 4). GnRHR2 was also identified by our laboratory in cell lines derived from porcine intestine and testis [Table 4; (48)]. Our group has also detected *GnRHR2* mRNA in various porcine tissues (e.g., testis, anterior pituitary, spleen, liver, large intestine, small intestine, and stomach) using conventional PCR (48).

### The Structure of GnRHR2

**Figure 4:**
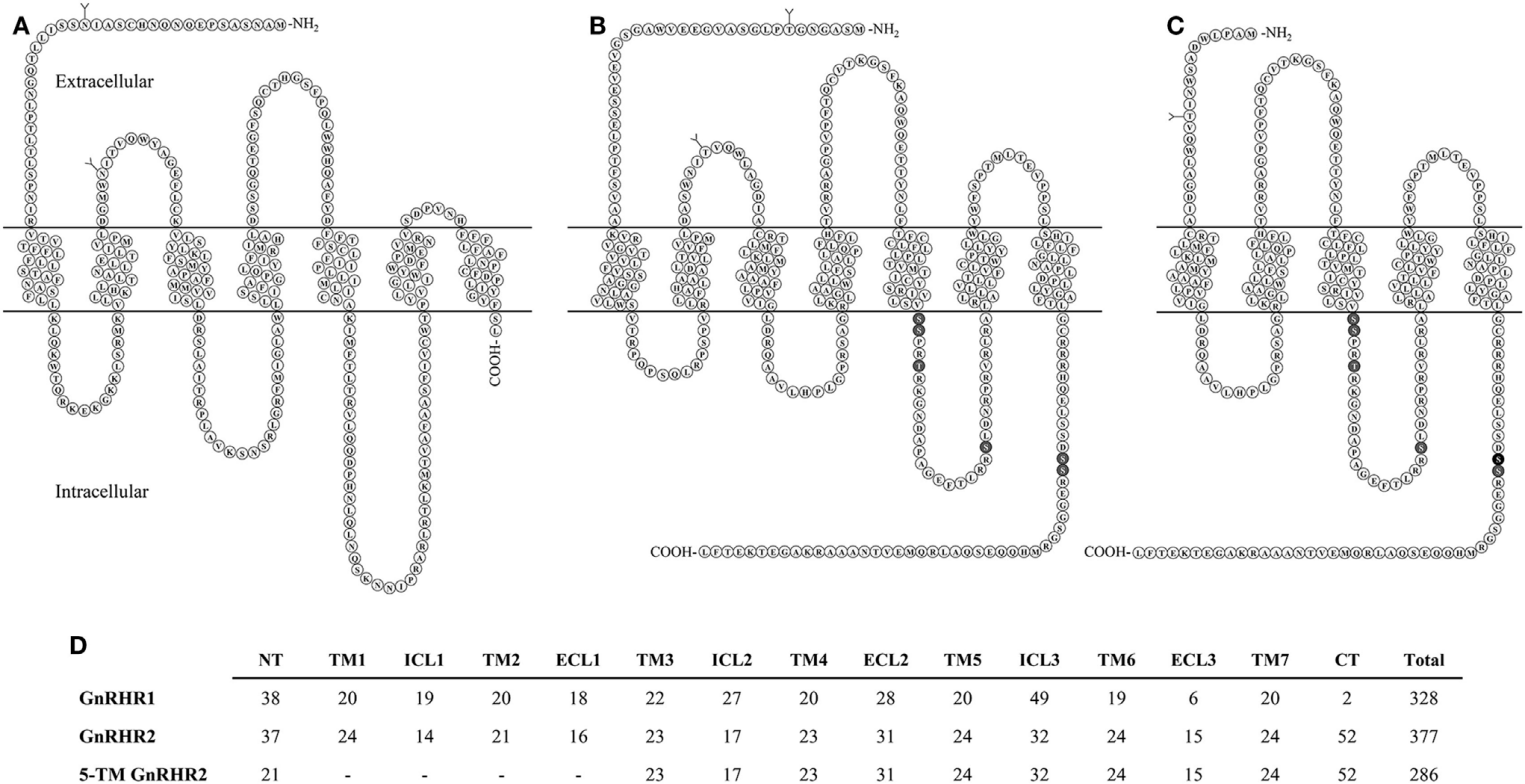
Structural comparison of GnRHRs in the pig. **(A)** GnRHR1 **(B)** GnRHR2 and **(C)** predicted 5-transmembrane (TM) GnRHR2 in the pig. GnRHR1 and GnRHR2 are both 7-TM G-protein coupled receptors; however, GnRHR1 lacks a C-terminal tail present in GnRHR2. The 5-TM GnRHR2 isoform lacks TM domains 1 and 2 due to alternative splicing in exon 1 of the *GnRHR2* gene, resulting in coupling of a truncated extracellular N-terminus directly to TM3. Glycosylation sites are represented by branched structures (Y) and the darkened residues in the GnRHR2 isoforms represent predicted sites of phosphorylation for internalization based on the work of Madziva et al. (102) and Ronacher et al. (103). Structures are predicted according to NCBI database records (GnRHR1, accession number NP_999438.1; GnRHR2, accession number AAS68622.1; 5-TM GnRHR2, accession number AAS68623.1). Adapted from Neill (80) and Neill et al. (82) with permission from Oxford University Press and Elsevier. **(D)** Comparison of residue number within each domain of GnRHR1, GnRHR2, and the predicted 5-TM GnRHR2 in the pig. Abbreviations: NT, extracellular N-terminus; TM, transmembrane; ICL, intracellular loop; ECL, extracellular loop; CT, C-terminal tail.

The full-length porcine GnRHR2 is 377 amino acids (NCBI accession number AAS68622.1) and has 42% homology to GnRHR1, which is 328 amino acids in length (Table 6; Figures 4A,B; NCBI accession number NP_999438.1). Like GnRHR1, the GnRHR2 is a member of the rhodopsin superfamily of GPCRs containing an extracellular N-terminus as well as seven TM α-helical domains connected *via* three ECLs and three ICLs [Figures 4A,B; (74)]. Differences in amino acid number within the domains of GnRHR1 and GnRHR2 are depicted in Figure 4D. Strikingly, GnRHR2 maintains a 52 amino acid C-terminal tail that is uniquely absent in GnRHR1 but similar to non-mammalian GnRHRs [Figures 4A,B; (74)]. Cytoplasmic tails are common among GPCRs and promote rapid receptor internalization and desensitization (100, 101). For example, the monkey GnRHR2 fully desensitized to GnRH2 treatment after 60 min whereas the tail-less human GnRHR1 failed to desensitize to a GnRHR1 agonist (Triptorelin) during the entire sampling period [90 min; (80)]. The cytoplasmic tail contributes to internalization differences between GnRHR1 and GnRHR2 as well. GnRHR1 is internalized without interacting with β-arrestin, whereas GnRHR2 can utilize β-arrestin for internalization (102), although it is not absolutely required (Table 6) (103). The phosphorylation of serine residues 338 and 339 in the C-terminus by GPCR kinases is critical for β-arrestin-independent internalization of GnRHR2 (103), whereas other regions within the C-terminal tail or ICL3 are sufficient for β-arrestin-dependent internalization (102). Therefore, two distinct pathways coordinate internalization of GnRHR2 (103). In addition, GnRHR2 internalization is dependent upon dynamin and likely mediated by both clathrin-coated pits and caveolae (103).

**Table 6:** Structural and functional characteristics of GnRHR1 and GnRHR2 in mammals.^a^

Characteristic	GnRHR2	GnRHR1	Reference
**Structure**			
Number of amino acids	377–380	327–328	(16, 74, 104)
5-transmembrane isoform	+	−	(80)
C-terminal tail	+	−	(16, 74, 104)
Amino acid conferring receptor activation in TM2 and TM7	Asp/Asp	Asn/Asp	(74, 105)
Amino acids conferring ligand selectivity in ECL3	Val–Pro–Pro–Ser	Leu–Ser–Asp/Glu–Pro	(106)

**Relative-binding affinities of native peptides^b^**			
GnRH1	1	15	(16)
GnRH2	24	1	(16)

**Relative activities of native peptides^c^**			
GnRH1	1	12	(16, 80)
GnRH2	90–440	1	(11, 16, 80)

**Relative activities of GnRHR agonists^c^**			
[d-Ala^6^] GnRH1	1	309	(80)
[d-Ala^6^] GnRH2	4	1	(104)
Buserilin	1	548	(80)
Triptorelin	1	395	(80)

**Relative activities of GnRHR antagonists^c^**			
Cetrorelix (SB-75)	1	>5,050	(80)
Trptorelix-1	1,660	1	(104)
Antide	1	>9,523	(80)
Antagonist 135-18	Full agonist	Full antagonist	(16)

**Coupling and signaling**			
G_αq/11_	+	+	(16)
Inositol phosphate (IP)	+	+	
Ca^2+^	+	+	(16, 50)
Protein kinase C	+	+	(16)
ERK 1/2	+ (sustained)	+ (transient)	(16)
p38 MAPK	+	−	(16)
c-Jun N-terminal kinase	−	−	(16)
Mammalian homolog r-Src of Rous sarcoma virus (c-Src)	−	+	(16)

**Receptor internalization**			
Rapid desensitization	+	−	(16)
Internalization rate	Rapid	Slow	(16)
β-Arrestin-dependent^d^	−	−	(102, 103, 107)
Dynamin dependent^e^	+	−/+	(103, 107)
Clathrin mediated	+	+	(103, 107)

*^a^Adapted from Millar (16) and Cheng and Leung (108)*.

*^b^Relative fold increase in affinity compared with the non-cognate ligand*.

*^c^Relative fold increase in activity compared with the non-cognate ligand or analog*.

*^d^Internalization of GnRHR2 can be mediated via β-arrestin but it is not required*.

*^e^GnRHR1 internalization is dependent on dynamin in the rat but not human*.

*Abbreviations: TM, transmembrane; ECL, extracellular loop; ERK 1/2, extracellular signal-regulated kinase 1 and 2; MAPK, mitogen-activated protein kinase*.

Other notable differences between the structure of GnRHR1 and GnRHR2 include alterations in residues that are highly conserved among GPCRs. GnRHR2 has an Asp/Asp microdomain in TM2 and 7, which is prevalent among GPCRs (74), whereas the GnRHR1 has a distinct Asn/Asp domain (Table 6). This region is thought to be important for receptor configuration and activation (105). Likewise, GnRHR2 has a divergent ligand-binding site (Val–Pro–Pro–Ser) within ECL3 compared with GnRHR1 (Leu–Ser–Asp/Glu–Pro; Table 6). This sequence probably determines the selectivity of GnRHR2 for GnRH2, as all other known ligand-binding sites are conserved between GnRHR1 and GnRHR2 (106). GnRHR2 and GnRHR1 also differ in the charge distributions of their extracellular domains, which may affect ligand binding (30).

### A 5-Transmembrane GnRHR2 Isoform

A 5-TM GnRHR2 isoform has been identified in pigs [Figure 4C; (109)]. Transcripts from this isoform are produced *via* alternative splicing in exon 1 and a different start codon. Therefore, TM1 and 2 are absent and the truncated extracellular N-terminus, comprised primarily of residues from ECL1, couples directly to TM3 [Figures 4C,D; (82)]. Transcripts for the 5-TM isoform were originally isolated from porcine pituitaries (109) but we have also found the 5-TM *GnRHR2* transcript in various tissues of the boar (e.g., testis, spleen, liver, large intestine, small intestine, and stomach) *via* conventional RT-PCR (48). The 5-TM transcript possesses all the characteristics of a translatable protein (109, 110) and would yield a 286 amino acid receptor (Figures 4C,D). Functionality of the 5-TM GnRHR2 has not been characterized thus far, but there is precedence for biologically active 5-TM GPCRs (111–114). As noted previously, the human may also produce a 5-TM *GnRHR2* transcript; however, it retains an in-frame premature stop codon (82).

## The Interaction Between GnRH2 and GnRHR2

### Functionality and Ligand Selectivity of GnRHR2

Receptor binding (29) and inositol phosphate (IP) accumulation (11, 29) assays established the selectivity of GnRH2 for GnRHR2. The binding affinity of GnRH2 for GnRHR2 is 24-fold greater than for GnRHR1 [Table 6; (29)]. When COS-1 cells overexpressing porcine *GnRHR2* cDNA were treated with GnRH2 or GnRH1, production of IP was stimulated with an EC_50_ of 0.5 nM for GnRH2 and 220 nM for GnRH1 (109). Conversely, the EC_50_ for GnRH2 binding the human GnRHR1 was 7.41 nM compared with 0.63 nM for GnRH1. The African green monkey (11) and marmoset GnRHR2 (29) were found to be functional and selective for GnRH2 as well. Thus, GnRH2 is 100- to 400-fold more active at the GnRHR2 than is GnRH1. In contrast, GnRH1 is only approximately 10-fold more active at GnRHR1 than is GnRH2 (Table 6).

Since GnRH1 and GnRH2 activate alternative GnRHRs, it became prudent to assess whether GnRH analogs are actually specific to their cognate receptors. However, it appears that GnRH1 and GnRH2 analogs, including GnRHR1 superagonists (Triptorelin and Buserilin), remain specific (Table 6) (80). At the GnRHR2, the native peptide (GnRH2) had an EC_50_ of 0.58 nM compared with 7.5 and 48 nM for Triptorelin and Buserilin, respectively. Therefore, GnRH2 is about 13- to 83-fold more potent at the GnRHR2 than are GnRHR1 superagonists (80). Non-native GnRH peptides (e.g., GnRH3) were considerably less potent (0.02%) than GnRH2 at eliciting IP production (80). Receptor specificity was also verified for GnRHR antagonists. Trptorelix-1 was identified as a GnRHR2-specific antagonist (104, 115), whereas Cetrorelix (also known as SB-75) and Antide are reported to be selective for GnRHR1 (80). For example, Antide mitigates both GnRH1- and GnRH2-induced IP production in cells overexpressing GnRHR1 but failed to ablate GnRH2-induced IP production in COS-7 cells overexpressing GnRHR2 (69). At elevated concentrations, however, Cetrorelix (SB-75) can non-specifically bind the GnRHR2 (50, 115). In addition, an established GnRHR1 antagonist (antagonist 135–18) was found to have agonistic properties at GnRHR2 (29). These data demonstrate the functionality and selectivity of the mammalian GnRHR2, but also indicate that both GnRHs can bind both mammalian receptors. However, GnRHR2 is highly selective for GnRH2, whereas GnRHR1 binds both ligands relatively well (16). Therefore, early studies examining the function of GnRH2 may be limited given that receptor binding was not addressed, and the actions of GnRH2 may have been inappropriately ascribed to GnRH1 (82).

### Cell Signaling of GnRHR2

Like GnRHR1, GnRHR2 couples to G_αq/11_ to initiate the production of IP, calcium mobilization, and the activation of protein kinase C (PKC; Table 6) (16, 84, 116). After the activation of PKC, however, GnRH1 and GnRH2 differentially stimulate mitogen-activated protein kinases (MAPKs) (29). GnRH1 transiently activated extracellular signal-regulated kinases 1/2 (ERK 1/2) and the proto-oncogene tyrosine-protein kinase, Src (c-Src) in COS-7 cells overexpressing GnRHR1 [Table 6; (29)]. Conversely, GnRH2 did not activate c-Src; instead, the interaction between GnRH2 and GnRHR2 activated ERK 1/2 in a prolonged manner as well as p38 MAPK in COS-7 cells overexpressing GnRHR2 (Table 6) (16, 29). Neither GnRH1 nor GnRH2 activated c-Jun N-terminal kinase (JNK) *via* their cognate receptors [Table 6; (29)]. Therefore, GnRHR1 and GnRHR2 exhibit differential signaling upon binding to their respective ligands. Moreover, activation of GnRHR1 by GnRH2 initiates different signaling pathways than GnRH1 (18). The seminal research uncovering the divergent signaling of GnRHR2 was conducted in transiently transfected cell lines (COS-7) by Millar et al. (29). More recent work utilizing immortalized human cancer cell lines has further explored GnRH2-induced signaling (50, 117–120). Although it is often unclear which receptor (GnRHR1 or GnRHR2) mediates the signal, because the presence of the GnRHR2 in humans is controversial (82). Another limitation is the use of cancer cells, which are inherently abnormal. Thus, there is a gap in our knowledge regarding the signaling cascades of GnRHR2 under normal physiologic conditions.

## Divergent Physiological Effects of GnRH2 Activating GnRHR1 and GnRHR2

### Gonadotropin Secretion

It was originally hypothesized that GnRH2 might function similarly to GnRH1 and elicit gonadotropin release from the anterior pituitary gland (12) or could be the much sought after FSH-releasing factor (16, 121–123). In support of these hypotheses, GnRH2 is present within regions of the brain (e.g., pre-optic and medio-basal hypothalamic areas) associated with the regulation of gonadotropin secretion (16). However, GnRH2 production in hypothalamic regions is scarce (33) and GnRH2 does not coexpress with GnRH1 in the hypothalamus (124). In contrast, GnRH2 is more highly abundant in other regions of the brain, such as the midbrain (28, 36, 37). Immunopositive GnRHR2 was detected on 69% of mammalian gonadotrope cells (29) but GnRH2 has never been isolated from hypothalamic portal blood (69, 125). Our laboratory detected immunoreactive GnRH2 in the hypothalamus of pigs, but abundance was low compared with the testis (34, 35). Millar et al. (29) identified GnRHR2 in the pituitary of the marmoset, although it was expressed at similar levels in numerous tissues unrelated to reproduction. It is now well established that GnRH2 and GnRHR2 are more highly expressed in peripheral tissues than the hypothalamus and anterior pituitary gland, respectively (20, 29, 33, 35), suggesting little role in gonadotropin secretion.

Upon its discovery, Miyamoto et al. (12) demonstrated that GnRH2 was less effective than GnRH1 at eliciting release of LH (68% less) and FSH (59% less) from pituitary cell cultures derived from rats. Other investigators confirmed these results in the rat and sheep through *in vitro* studies (125–127). GnRH2 was 92% less effective than GnRH1 at stimulating gonadotropin secretion from primary cultures of ovine pituitary cells (126). *In vivo*, a bolus (10 µg) of GnRH2-stimulated LH and FSH release in rams, although less robustly (40-fold) than GnRH1. There was a modest preference (2-fold) for FSH over LH secretion in response to treatment with GnRH2 (29). In the rat (121) and rhesus macaque (128), however, GnRH2 did not preferentially stimulate FSH release compared with GnRH1.

The effects of GnRH2 on gonadotropin secretion in rats and sheep are likely mediated through the GnRHR1, because both species lack GnRHR2 (21) and GnRHR1 can be activated by GnRH2, although with 10-fold less activity than GnRH1 (29). Indeed, a GnRHR1-specific antagonist completely blocked both chronic and acute GnRH2-stimulated secretion of gonadotropins in sheep (69) as well as in pituitary cell cultures that were derived from rats (127). These data provide strong evidence that GnRH2 is a weak stimulator of gonadotropin secretion in mammals *via* interaction with GnRHR1, although it remains plausible that this interaction, albeit minimal, may still be physiologically relevant (80). For example, it has been suggested that GnRH2 primes activity and/or production of GnRHR1. However, cotreatment of monkey pituitary cells (125, 128) and rams (69) with GnRH2 and GnRH1 did not enhance LH or FSH secretion above GnRH1 treatment alone. Alternatively, Urbanski (129) proposed that GnRH2 activates GnRHR1 to mediate the preovulatory LH surge. However, this hypothesis has not yet been evaluated *in vivo*.

The effect of GnRH2 on secretion of LH and FSH in species that produce a functional GnRHR2 has also been examined. Treating musk shrews with GnRH2-stimulated ovulation, with 10-fold less potency than GnRH1, but this effect could be blocked with a GnRHR1 antagonist (33). Treating rhesus macaques with GnRH2 elicited increased secretion of gonadotropins *in vivo* during the follicular and luteal phase of the menstrual cycle, but the response was not compared with GnRH1 (37). Others showed that a high dose (1 µg/kg of body weight) of GnRH1 and GnRH2 were equipotent at stimulating release of LH and FSH in the female rhesus macaque (128). However, in cultures of pituitary cells derived from male rhesus macaques, GnRH2 was a less effective stimulator of gonadotropin secretion than GnRH1 (125). GnRH2 stimulated secretion of LH with an EC_50_ of 0.37 nM (compared with 0.10 nM for GnRH1) and FSH with an EC_50_ of 0.59 nM (versus 0.10 nM for GnRH1) (125). Receptor antagonism was used to clarify if GnRHR1 or GnRHR2 mediated these effects. Interestingly, GnRH2-induced gonadotropin secretion was completely blocked by treatment with Antide (125, 128), a GnRHR1-specific antagonist that has minimal activity (EC_50_ of 10,000 nM) at the GnRHR2 (80). Thus, the stimulatory effects of GnRH2 on gonadotropin secretion were attributed to its interaction with GnRHR1.

Similar results were observed in the pig; Cetrorelix (GnRHR1 antagonist) mitigated GnRH2-induced LH and FSH secretion from porcine gonadotrope cell cultures (109). Data from our laboratory support these results. Treatment of boars with [d-Ala^6^] GnRH2 weakly stimulated secretion of LH compared with [d-Ala^6^] GnRH1 (35). In addition, treatment of males with a GnRHR2-specific antagonist (Trptorelix-1) failed to suppress LH secretion (35). Likewise, immunization of boars against GnRH2 did not affect gonadotropin secretion compared with control males (130). Finally, LH secretion was unaffected in transgenic swine with ubiquitous knockdown of GnRHR2 (53). Collectively, these data demonstrate that only high doses of GnRH2 can elicit weak gonadotropin release *via* the GnRHR1. Therefore, GnRH2 and its receptor do not appear to be physiological stimulators of gonadotropin secretion in mammals.

### Reproductive Behavior and Energy Balance

Given the neuroanatomical location of GnRH2 (e.g., midbrain and limbic structures), investigators hypothesized that it may be important in sexual behavior (14, 29, 131). The first evidence demonstrating a role for GnRH2 in female reproductive behavior occurred when GnRH2, but not GnRH1, infusions into the brain of female sparrows increased receptivity to songs of male sparrows (132). In mammals, the effects of GnRH2 on sexual behavior may be dependent on metabolic state. When feed was restricted by 60%, female musk shrews displayed fewer sexual behaviors (31) and had reduced *GnRH2* mRNA expression (midbrain GnRH2 cells) and protein abundance (ventromedial nucleus, medial habenula, GnRH2 cells, and midbrain central gray) compared with *ad libitum* fed animals (133). Sexual behaviors as well as *GnRH2* mRNA expression (midbrain) and protein levels in some regions (ventromedial nucleus and medial habenula) returned to normal after only 90 min of *ad libitum* feeding after restriction (133). The lordosis response in mice during nutrient restriction was enhanced by GnRH2, but not GnRH1 (134); no effect of GnRH2 on lordosis was observed when female mice were fed *ad libitum*. Mice lack GnRH2 and GnRHR2 (30), therefore the biological significance of these results is unclear. In male musk shrews, GnRH2 abundance was examined in all regions of the midbrain prior to and after feed restriction. Unlike females, GnRH2 abundance and sexual behaviors were not affected by feed restriction in male musk shrews. This is potentially due to sexually dimorphic expression patterns of GnRH2 between male and female musk shrews or the level of feed restriction (133, 135).

The enhancement of sexual behaviors in female musk shrews treated with GnRH2 during feed restriction were not attenuated by the addition of a GnRHR1 antagonist (Antide), suggesting the effect of GnRH2 on sexual behavior is mediated *via* the GnRHR2 (32). Similarly, reproductive behaviors of female musk shrews during food restriction were rescued by treatment with antagonist 135–18 (32), which simultaneously acts as an antagonist of GnRHR1 and an agonist of GnRHR2 (29). This agrees with results in female marmoset monkeys showing that GnRH2 and antagonist 135–18, but not GnRH1, increased proceptive (sexual solicitation) behaviors (136). In contrast, other studies showed that high doses (25 µg) of GnRH1 do elicit sexual proceptivity of female marmosets (137), but these effects are likely mediated by GnRHR2 (136).

The interaction between metabolic state and the effects of GnRH2 on sexual behavior may be related to the potential effects of GnRH2 on the mechanism of food intake. Intracerebroventricular infusions of GnRH2 reduced feed intake (33%) in female musk shrews that were underfed (138). A similar reduction (28%) in food intake of musk shrews was also apparent with GnRH2 treatment during *ad libitum* feeding (138). The ability of GnRH2 to reduce food intake was acute, beginning 90 min after GnRH2 infusion and persisting for 3 h, regardless of nutritional plane (138). The effect of GnRH2 on food intake is probably mediated through the GnRHR2 as treatment with Antide (GnRHR1 antagonist) did not prevent a GnRH2-induced reduction in feed intake (32).

The results of these studies demonstrate that GnRH2 influences female reproductive behavior through the GnRHR2, not GnRHR1. GnRH2 may be acting as a permissive neuropeptide that links reproductive behavior with nutritional status in some species (32, 136). If energy balance is low, GnRH2 production is decreased, inhibiting reproductive behaviors and increasing feed intake. If energy is abundant, increased GnRH2 expression will promote mating behaviors (32).

### Testicular Function

For 35 years, evidence has been accumulating that GnRH1 and GnRHR1 have extrapituitary functions. Both GnRH1 and GnRHR1 are expressed within the testes of some mammals (e.g., humans, rats, and mice) (139–144) and their interaction directly elicits testosterone secretion from Leydig cells (145–148). The contribution of GnRH1 and its receptor to localized control of gonadal function is not well understood and therefore often overlooked (149). Localized regulation of the gonads by GnRHs may have existed early in evolution to control reproduction before the formation of an organized pituitary gland (7). For instance, GnRH peptides control reproductive function of invertebrates (e.g., mollusks, echinoderms, and prochordates) that lack an anterior pituitary gland (150–152). *Ciona intestinalis* synthesizes GnRH1 and GnRH2 directly within its gonads and treating gonadal cultures with GnRH1 and GnRH2 stimulates secretion of sex steroids (153). Moreover, seven novel tunicate GnRHs stimulated the release of gametes in *C. intestinalis*, indicative of direct action on the gonads (152). Local control of gonadal function may have been retained in mammals for a specific purpose not met by the gonadotropins. McGuire and Bentley (149) proposed that function might be a rapid, transient responsiveness to environmental stimuli.

**Figure 5:**
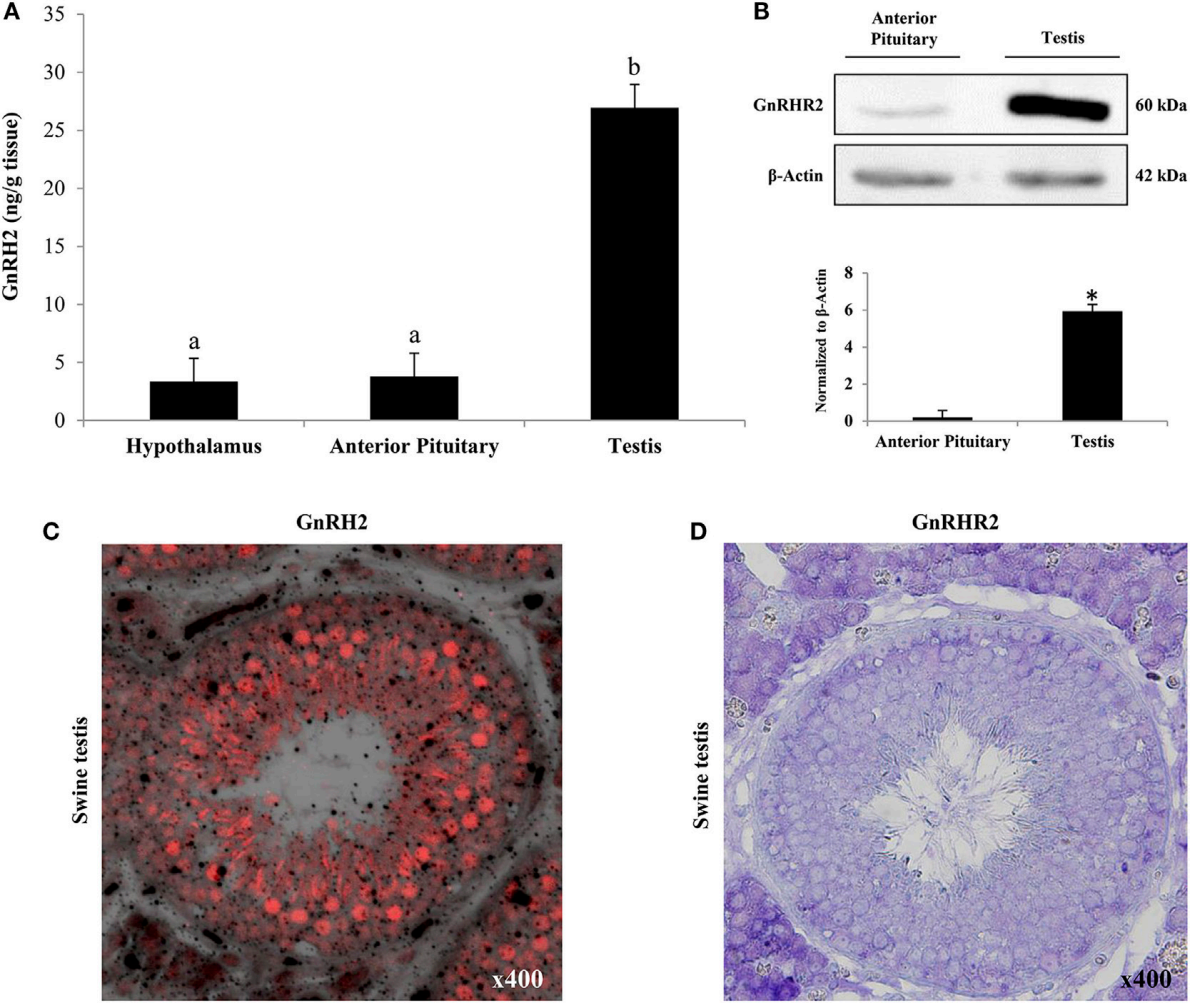
Both GnRH2 and GnRHR2 are abundantly produced within the porcine testis. **(A)** GnRH2 levels were determined *via* ELISA of homogenates from the hypothalamus, anterior pituitary gland, and testis of males (*n* = 6). ^a,b^Bars with alternate letters differ (*P* < 0.0001). **(B)** Representative western blot of anterior pituitary gland and testicular tissue from boars (*n* = 5) using an antibody directed against GnRHR2 (upper panel). Immunoblot quantification revealed a difference in GnRHR2 protein levels between tissue types (*P < 0.0001; lower panel). **(C)** GnRH2 is primarily localized to the tubular compartment of the porcine testis. Representative immunofluorescence image (400×; merge of fluorescence and transmitted light) of porcine testes (*n* = 5) using an antibody directed against GnRH2 (red). **(D)** GnRHR2 localizes to both the tubular and interstitial compartments of the porcine testis. Representative immunohistochemistry image (400×) of porcine testes (*n* = 7) using an antibody directed against GnRHR2 (purple). Reprinted from Desaulniers et al. (35) with permission from Oxford University Press.

**Figure 6:**
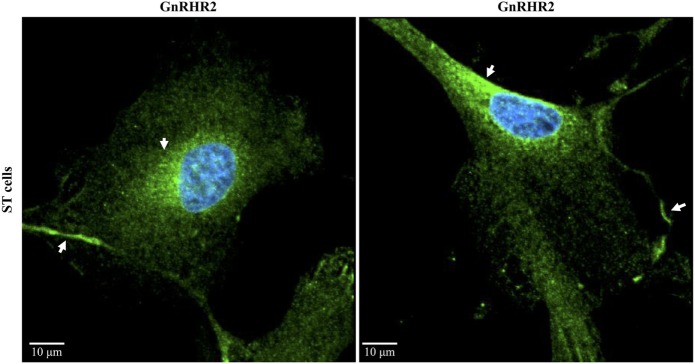
Subcellular localization of the GnRHR2 in a swine testis (ST)-derived cell line. Immunocytochemistry was performed on ST cells (CRL-1746; American Type Culture Collection, Rockville, MD, USA) with an antibody directed against GnRHR2 (1:100–1:200; sc-162889; Santa Cruz Biotechnology, Santa Cruz, CA, USA) and labeled with an Alexa Fluor 488 secondary antibody (green); nuclei were stained with DAPI (blue). The experiment was replicated three times. Two representative confocal microscopy images are shown. Note the plasma membrane and perinuclear staining (arrows). The scale bar represents 10 µm. Adapted from Cederberg et al. (48).

Of the 31 organs examined in the marmoset monkey, *GnRHR2* transcript levels were most abundant in the testis (29). In fact, several laboratories have now reported the presence of GnRH2 and/or its receptor within mammalian testes (11, 20, 29, 35, 46, 154), suggesting an autocrine/paracrine role in testicular function. Abundance of GnRH2 in the testis of the pig was 7-fold greater than levels within the anterior pituitary gland or hypothalamus (Figure 5A) (35). This corresponded to 6-fold more GnRHR2 protein in the testis than in the anterior pituitary gland (Figure 5B). We also observed the most intense GnRH2 immunostaining within the seminiferous tubules of the boar, primarily localizing to germ and Sertoli cells (Figure 5C) (35), although some signal was present within the interstitium. Likewise, we have detected immunoreactive GnRHR2 on germ and Sertoli cells as well as the plasma membrane of porcine Leydig cells (Figure 5D). Subcellular localization of GnRHR2 in immortalized ST-derived cells, recently shown to exhibit Sertoli cell-like properties (155), revealed plasma membrane as well as perinuclear immunostaining (Figure 6) (48). These data establish that GnRH2 and its receptor are abundantly produced in the porcine testis, indicating an important autocrine/paracrine role in testis biology.

The first association of GnRH2 with testicular function was reported in humans. Although the levels of *GnRHR2* mRNA were not evaluated, testicular *GnRH1, GnRH2*, and *GnRHR1* transcript abundance was increased in infertile (azoospermic) men, corresponding with elevated intra-testicular testosterone levels and increased expression of genes encoding steroidogenic enzymes (*CYP11A1* and *HSD3B*) (154). Notably, *GnRH1* and *GnRH2* mRNA levels were positively correlated with expression of *HSD3B*, intra-testicular testosterone levels, and concentrations of FSH in serum, indicating that the testicular GnRH system helps regulate spermatogenesis and steroidogenesis in humans (154). Consistent with this, *GnRHR2* transcripts have been detected in post-meiotic germ cells and human sperm (46). Our laboratory has immunolocalized GnRHR2 to ejaculated porcine spermatozoa, implying a role for GnRH2 in sperm function of boars (34, 45). In contrast, it was concluded that GnRH2 did not impact spermatogenesis in mice (156); however, mice lack both GnRH2 and its receptor (21), which limits the interpretation of these results.

Based upon the aforementioned discovery of GnRHR2 on porcine Leydig cells (35), our laboratory became interested in whether GnRH2 and its receptor are autocrine/paracrine regulators of steroidogenesis in the pig. Previous research has indirectly revealed a role for GnRH2 and its receptor within the testes of mature swine. For example, testosterone secretion was reduced in males immunized against GnRH2, but concentrations of LH in serum remained unchanged (130). Primary cultures of Leydig cells from boars immunized against GnRH2 demonstrated impaired secretion of testosterone basally and when stimulated with LH (130). In a different study, treatment of males with a GnRHR1 antagonist (SB-75; Cetrorelix) blunted hCG-induced secretion of testosterone (157). In a subsequent trial, release of testosterone in boars was continuously reduced during chronic administration of SB-75, yet secretion of LH was only transiently suppressed (158). In addition, SB-75 attenuated hCG-stimulated secretion of testosterone from porcine testicular explants (158). These data imply that a testicular GnRHR was directly regulating steroidogenesis locally within the swine testis. Given that GnRHR1 is not expressed within the porcine testis (158), the results of these studies may be ascribed to GnRHR2. Therefore, we hypothesized that GnRH2, produced locally in the testis, binds to the GnRHR2 on porcine Leydig cells to stimulate LH-independent secretion of testosterone.

**Figure 7:**
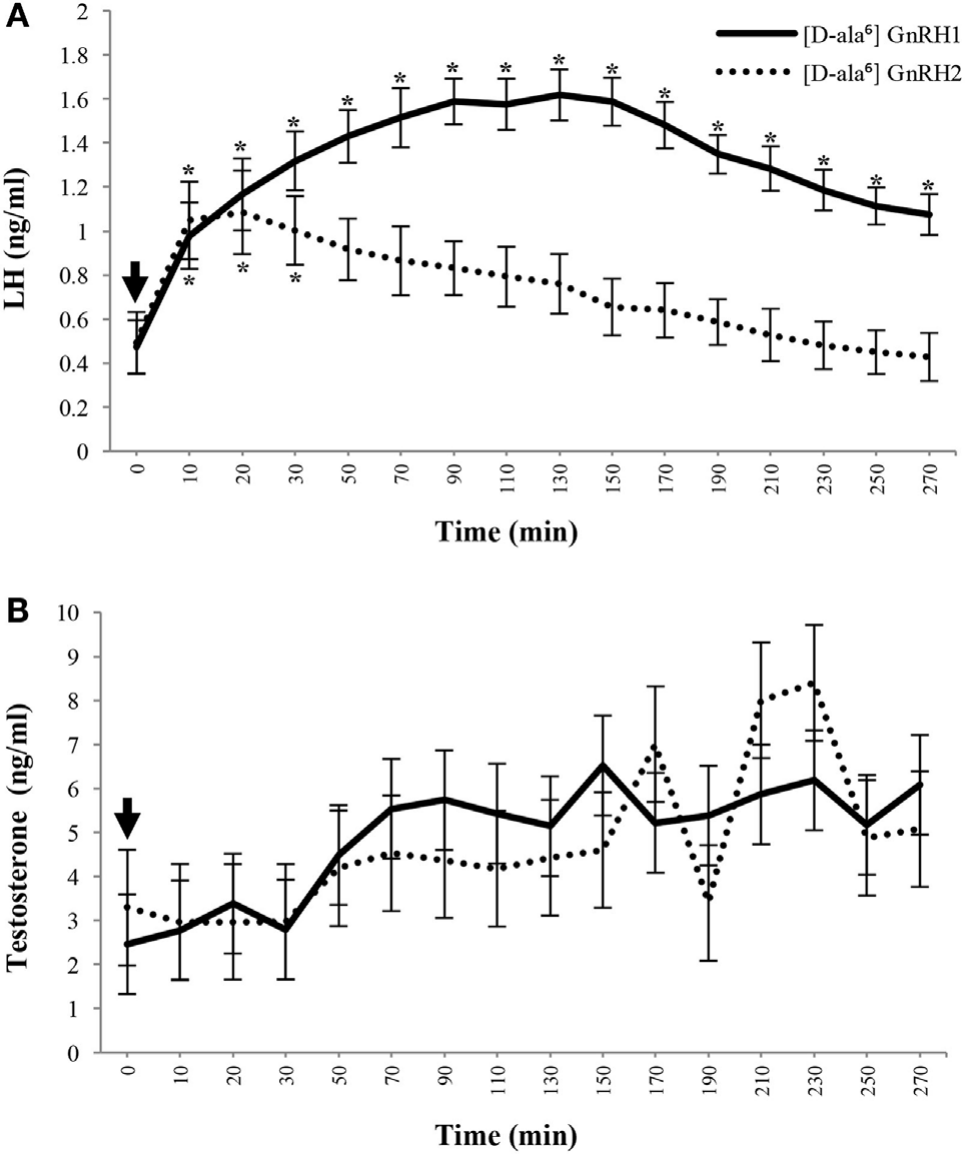
GnRH2 stimulates secretion of testosterone similar to GnRH1 despite reduced luteinizing hormone (LH) release in mature male pigs. Plasma concentrations of LH **(A)** and testosterone **(B)** after intravenous administration of [d-ala^6^] GnRH1 (solid line; *n* = 8) or [d-ala^6^] GnRH2 (dotted line; *n* = 6) to cannulated adult males. After pretreatment sampling, treatments were administered (arrow; 0 min) and blood was serially collected. Data are presented as the Least Squares Means (±SEM). For LH, Treatment, *P* < 0.001; Time, *P* < 0.0001; Treatment × Time, *P* < 0.03. For testosterone, Treatment, *P* = 0.1929; Time, *P* < 0.002; Treatment × Time, *P* = 0.7190. *Within treatment, concentrations differ from pre-injection levels (*P* < 0.05). GnRH1 differences are indicated above error bars and GnRH2 differences are labeled below error bars. Reprinted from Desaulniers et al. (35) with permission from Oxford University Press.

To test this hypothesis, we treated porcine testicular explants with hCG in the presence or absence of GnRH2. Secretion of testosterone was significantly stimulated after treatment with GnRH2 or hCG but there was no synergistic effect of treating with hCG and GnRH2 (35). These results established that GnRH2 was a stimulator of acute testosterone secretion *ex vivo*. We next tested the effect of GnRH2 *in vivo*. White crossbred boars were fit with indwelling jugular cannulae to perform serial bleeding trials following treatment with GnRH analogs. GnRH2 infusion robustly elicited testosterone secretion, similar to GnRH1 treatment, despite minimal LH secretion when compared with GnRH1-stimulated males [Figure 7; (35)]. Furthermore, GnRH2-induced secretion of testosterone was blunted by pretreatment with the GnRHR1 antagonist, SB-75 (35), which can antagonize GnRHR2 (50, 115). We hypothesize that SB-75 reduced GnRH2-induced testosterone secretion by antagonizing GnRHR2 directly in the testis because the secretory pattern of LH was unaffected compared to trials where only GnRH2 treatments were administered (35). Finally, intratesticular injections of either GnRH1 or GnRH2 stimulated secretion of testosterone compared with saline-treated controls; however, GnRH2 did so without eliciting the release of LH, unlike GnRH1 (159). Together, these data support our hypothesis that GnRH2 is stimulating testosterone production directly at the testis in the absence of the classical androgen regulator, LH.

**Figure 8:**
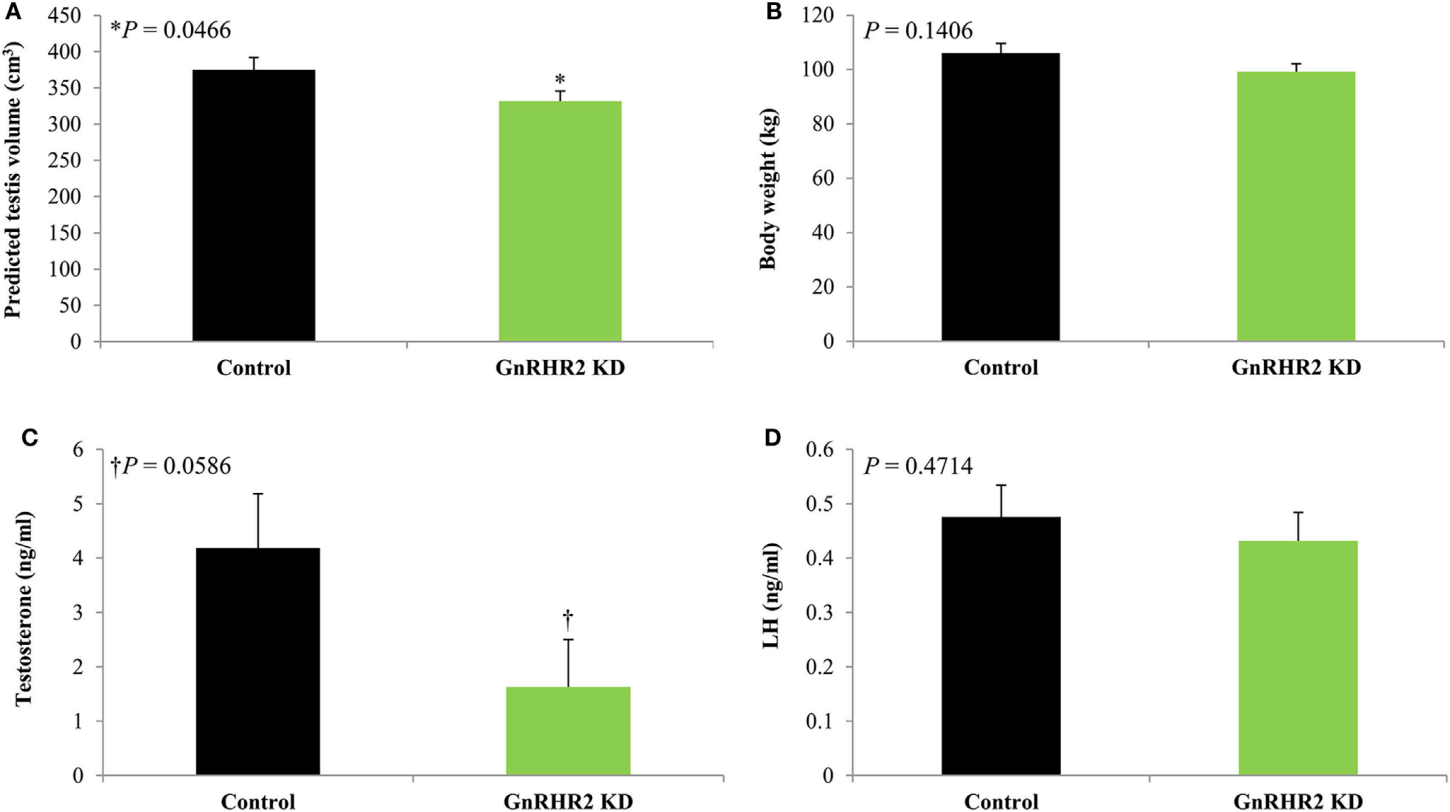
GnRHR2 knockdown (KD) swine have smaller testes and reduced serum testosterone concentrations but similar body weights and circulating luteinizing hormone (LH) levels compared with littermate control animals during pubertal development. At 40, 100, 150, 190, 225, and 300 days of age, blood was collected from GnRHR2 knockdown (*n* = 10) and littermate control (*n* = 7) males; at the same time points, body weight and predicted testis volume were measured. Serum testosterone and LH concentrations were determined by radioimmunoassay. Data are presented as the Least Squares Means (±SEM). The *P*-value for the main effect of line is indicated. **(A)** Predicted testis volume, **(B)** body weight, **(C)** serum testosterone concentrations, and **(D)** serum LH concentrations. Adapted from Desaulniers et al. (53); no permission is required from the copyright holder.

To further study the role of GnRH2 and its receptor in pigs, our laboratory generated a GnRHR2 knockdown swine line (53). These animals ubiquitously express short hairpin RNA targeting the porcine GnRHR2. Consequently, testicular *GnRHR2* mRNA levels were reduced by 70% in adult males compared with littermate control animals (53). During pubertal development, GnRHR2 knockdown boars had smaller testes (Figure 8A) despite a normal body weight (Figure 8B), implying impaired testicular function. Moreover, testosterone concentrations tended to be lower (Figure 8C) in transgenic versus littermate control males, yet LH concentrations were unaffected [Figure 8D; (53)]. These results support our hypothesis that activation of GnRHR2 on porcine Leydig cells stimulates LH-independent testosterone secretion. These swine represent the first genetically engineered animal model to study the function of GnRH2 and its receptor in mammals and are currently being utilized to identify the molecular mechanisms linking GnRHR2 and steroidogenesis in male pigs. Given that testosterone and its metabolites govern male fertility (e.g., sex differentiation, reproductive tract maintenance, libido, spermatogenesis, and accessory sex gland function), GnRH2 and its receptor are novel molecular targets to enhance reproductive efficiency in swine.

### Female Reproduction

Although GnRH2 and its receptor are expressed within female reproductive tissues (Table 3), few studies have directly examined the role of the GnRH2/GnRHR2 system in the female. Several lines of evidence suggest that GnRH2 and its receptor are novel regulators of placental function, implantation, and ovarian steroidogenesis. GnRH2 is produced by the human placenta in a pulsatile manner and is six times more stable than GnRH1 when exposed to placental enzymes (70). GnRH2 stimulated the production of hCG in human placental explants (70, 160–162), whereas GnRH1 treatment did not (70). Moreover, a high affinity receptor specific for GnRH2 (70), as well as immunoreactive GnRHR2 (163), have been detected in the human placenta.

When compared with GnRH1, GnRH2 potently enhanced invasion of human trophoblasts (84) through extracellular matrix remodeling (164). Notably, antagonizing or knocking down GnRHR1 abrogated GnRH1-mediated effects on trophoblast cells but did not influence GnRH2-stimulated invasion (84, 165, 166). In addition, GnRH2 (mRNA and protein) was found in the uterine endometrium (stromal and glandular epithelial cells) of women during all phases of the menstrual cycle, although GnRH2 production increased during the secretory phase, indicative of a role in implantation (67). The GnRHR2 may be produced in human endometrium as well given that both high and low affinity-binding sites for GnRH1 were identified in human endometrial cancer cells (167). Given that GnRH1 binds GnRHR2 with 15-fold less affinity (29), the low affinity-binding site for GnRH1 detected in this study may be the GnRHR2. Indeed, others have demonstrated that immunoreactive GnRHR2 is present in human endometrial adenocarcinomas (163). Treatment of rhesus macaques with a GnRHR2 agonist near ovulation conferred contraceptive actions in 100% of females; this effect was not mediated by the inhibition of progesterone secretion (168). In a subsequent study, pregnancy was prevented in all females receiving GnRH2 treatment, whereas 62.5% of saline-treated controls became pregnant. Interestingly, the low doses of GnRH2 (2–8 μg/day) inhibited secretion of progesterone, whereas high doses (16–32 μg/day) had no effect (169). Nevertheless, pregnancy was prevented in all treatment groups, indicating that the contraceptive activity of GnRH2 is not confined to the suppression of progesterone production alone.

In other species, GnRH2 appears to modulate secretion of progesterone as well. Kang et al. (58) reported that GnRH2 inhibited basal and hCG-stimulated progesterone secretion from human granulosa-luteal cells. In the baboon, GnRH2 is present in the ovary and released from granulosa cells *in vitro*. Exogenous GnRH2 administration suppressed production of progesterone from cultured granulosa cells by 75% (60), whereas GnRH1 failed to suppress progesterone release. Binding kinetics indicated two binding sites for GnRH2, a high and a low affinity site, compared with only one binding site for GnRH1, suggesting the presence of a GnRH2-specific receptor in the baboon ovary (60). Collectively, these data are compelling; however, more studies are needed to determine how GnRH2 and its receptor impact female reproduction in mammals. Toward this end, research in our laboratory is currently underway to define the role of GnRH2 and its receptor in reproductive function of the female pig.

### Cancer

It is well established that GnRH1 and its receptor are expressed in cancer cells derived from reproductive tissues and administration of GnRH1 analogs inhibits their proliferation (51, 170, 171). GnRH2 and its receptor may also influence the progression of reproductive cancers, given that both are expressed in cancer cells (Table 4) and tumors derived from reproductive tissues (38, 44, 51, 170, 171). Growing evidence indicates that treatment of cancer cells with GnRH2 analogs inhibits their proliferation. This has been demonstrated in prostate (54, 172), ovarian, breast, and endometrial cancer cells (38, 40, 170, 171). Interestingly, GnRH2 appears to have a more potent anti-proliferative effect than GnRH1 (51). The cellular mechanisms by which GnRH2 mediates this effect have been the subject of recent research efforts. Treatment of breast cancer cells with either GnRH1 or GnRH2 inhibited ribosomal phosphoproteins, which are needed for proper protein translation and cell proliferation (38). GnRH2 also increased metalloproteinase production, key regulators of tumor invasion, in ovarian cancer cells (173). In addition, treatment with a GnRH2 agonist reduced cell proliferation and inhibited the mitogenic effects of epidermal growth factor in human endometrial and ovarian cancer cells (120). In addition, GnRH2 and its receptor may have emerging roles in the modulation of cell proliferation *via* extracellular vesicles. For example, glioblastoma-derived microvesicles increased proliferation of tumor cells *in vitro*; the same microvesicles were also found to carry *GnRH2, GnRH1, GnRHR2*, and *GnRHR1* mRNA (174). Thus, GnRH/GnRHR transcripts packaged in extracellular vesicles could be an unexploited mechanism to affect tumor progression in humans.

In addition to anti-proliferative actions, GnRH2 analogs may also exert pro-apoptotic effects on cancer cells. Treatment with several GnRHR2 antagonists inhibited growth of human endometrial and ovarian cancer cells *in vitro* and *in vivo via* caspase 3-dependent mechanisms (175); a different GnRHR2 antagonist (SN09-2) induced apoptosis in prostate cancer cells (172). Likewise, GnRH2 can increase apoptosis *via* the caspase-dependent pathway in human granulosa cells (176). In breast cancer cells, yet another GnRHR2 antagonist induced apoptotic cell death *in vitro* and *in vivo* (177). In addition to anti-proliferative and pro-apoptotic effects, GnRH2 might also regulate cellular autophagy. Human prostate cancer cells treated with a GnRHR2 antagonist (Trptorelix-1) displayed increased mitochondrial dysfunction as well as autophagosome formation (178). These cells had decreased Akt phosphorylation and increased c-Jun phosphorylation, additional hallmarks of cell autophagy. In numerous cases, the effects of GnRH2 on cancer cells were not mediated through the GnRHR1, implying a role for human GnRHR2 (50, 52, 120, 177). Thus, GnRH2 and possibly its receptor, regulate cancer cell proliferation/survival and represent emerging targets for the development of new cancer therapies.

## Conclusion

GnRHs are ancient peptides which may have first functioned to directly regulate the gonads before evolving into specialized modulators of gonadotropin secretion. GnRH2 is the most ancient of the GnRHs and has been completely conserved from boney fish to man, signifying a critical biological role. Moreover, GnRH2 is structurally unique from GnRH1, which promotes its stability and half-life. A highly selective receptor specific for GnRH2 is also produced in mammals. The GnRHR2 is dissimilar from GnRHR1, containing an intracytoplasmic tail and eliciting divergent cell signaling cascades. The genes for *GnRH2* and/or *GnRHR2* have been deleted or inactivated in many species, but both are functional in old world monkeys, musk shrews and pigs, implying an essential role in these animals. Moreover, evidence continues to suggest the presence of GnRHR2 in humans despite apparent coding errors in the gene. Data from numerous species (including humans) demonstrates that GnRH2 and its receptor are ubiquitously expressed. Notably, both are produced in low abundance in regions of the brain associated with gonadotropin secretion and highly expressed in peripheral reproductive organs. Thus, GnRH2 and its receptor are both structurally and functionally distinct from their classical counterparts.

Contrary to their name, GnRH2 and GnRHR2 are not physiological stimulators of gonadotropin secretion in mammals. Instead, GnRH2 and its receptor have been implicated in various functions, mostly pertaining to mammalian reproduction. The first defined function of GnRH2 and its receptor was the modulation of sexual behavior, based on nutritional status, in females. Within peripheral tissues, GnRH2 and its receptor are important regulators of reproductive organs in both males and females. For example, GnRH2 and its receptor directly regulate steroidogenesis within the porcine testis. In the female, GnRH2 and its receptor may mediate placental function, implantation, and ovarian steroidogenesis. Furthermore, both *GnRH2* and *GnRHR2* are expressed in human reproductive tumors and are emerging targets for cancer treatment. Therefore, GnRH2 and its receptor are critical modulators of reproductive function in mammals, albeit *via* a divergent mechanism from the classical GnRH1 and GnRHR1 interaction. More work is needed to better understand the importance of localized regulation by the GnRH2/GnRHR2 system, but its contribution to mammalian reproduction is unequivocal.

Despite these data, the ubiquitous nature of GnRH2 and GnRHR2 suggests that many more biological functions remain undefined. The deletion of the *GnRH2* and *GnRHR2* genes from the rat and mouse has undoubtedly inhibited the study of this system in a widely available and economical laboratory animal. However, the recent development of a GnRHR2 knockdown swine line provides an essential animal model to explore the functions of GnRH2 and its receptor in mammals. Given the recent discovery of the *GnRH2* and *GnRHR2* genes in numerous mammalian species, this system may be physiologically relevant and unexploited in a wide range of species.

## Author Contributions

AD and BW determined the structure and content of this review. AD performed the literature review and composed the manuscript with input and assistance from RC, CL, and BW. RC and AD developed the figures/tables. AD, RC, CL, and BW edited and revised the manuscript.

## Conflict of Interest Statement

The authors declare that there is no conflict of interest to disclose. Mention of trade names or commercial products in this publication is solely for the purpose of providing specific information and does not imply recommendation or endorsement by the U.S. Department of Agriculture. The U.S. Department of Agriculture (USDA) prohibits discrimination in all its programs and activities on the basis of race, color, national origin, age, disability, and where applicable, sex, marital status, familial status, parental status, religion, sexual orientation, genetic information, political beliefs, reprisal, or because all or part of an individual’s income is derived from any public assistance program. (Not all prohibited bases apply to all programs.) Persons with disabilities who require alternative means for communication of program information (Braille, large print, audiotape, etc.) should contact USDA’s TARGET Center at (202) 720-2600 (voice and TDD). To file a complaint of discrimination, write to USDA, Director, Office of Civil Rights, 1400 Independence Avenue, S.W., Washington, D.C. 20250-9410, or call (800) 795-3272 (voice) or (202) 720-6382 (TDD). USDA is an equal opportunity provider and employer.

## References

[1] Mason AJ Hayflick JS Zoeller RT Young WS III Phillips HS Nikolics K A deletion truncating the gonadotropin-releasing hormone gene is responsible for hypogonadism in the hpg mouse. Science (1986) 234:1366–71. 10.1126/science.3024317 3024317

[2] Schwanzel-Fukuda M Bick D Pfaff DW. Luteinizing hormone-releasing hormone (LHRH)-expressing cells do not migrate normally in an inherited hypogonadal (Kallmann) syndrome. Brain Res Mol Brain Res (1989) 6(4):311–26. 10.1016/0169-328X(89)90076-4 2687610

[3] Charlton HM Halpin DM Iddon C Rosie R Levy G McDowell IF The effects of daily administration of single and multiple injections of gonadotropin-releasing hormone on pituitary and gonadal function in the hypogonadal (hpg) mouse. Endocrinology (1983) 113(2):535–44. 10.1210/endo-113-2-535 6409585

[4] Schally AV Arimura A Baba Y Nair RM Matsuo H Redding TW Isolation and properties of the FSH and LH-releasing hormone. Biochem Biophys Res Commun (1971) 43(2):393–9. 10.1016/0006-291X(71)90766-2 4930860

[5] Matsuo H Baba Y Nair RM Arimura A Schally AV Structure of the porcine LH- and FSH-releasing hormone. I. The proposed amino acid sequence. Biochem Biophys Res Commun (1971) 43(6):1334–9. 10.1016/S0006-291X(71)80019-0 4936338

[6] Baba Y Matsuo H Schally AV Structure of the porcine LH- and FSH-releasing hormone. II. Confirmation of the proposed structure by conventional sequential analyses. Biochem Biophys Res Commun (1971) 44(2):459–63. 10.1016/0006-291X(71)90623-1 4946067

[7] Millar RP. GnRHs and GnRH receptors. Anim Reprod Sci (2005) 88(1–2):5–28. 10.1016/j.anireprosci.2005.05.032 16140177

[8] Dubois EA Zandbergen MA Peute J Goos HJ Evolutionary development of three gonadotropin-releasing hormone (GnRH) systems in vertebrates. Brain Res Bull (2002) 57(3–4):413–8. 10.1016/S0361-9230(01)00676-1 11923000

[9] Sherwood NM The GnRH family of peptides. Trends Neurosci (1987) 10(3):129–32. 10.1016/0166-2236(87)90058-0

[10] Sower SA Chiang YC Lovas S Conlon JM. Primary structure and biological activity of a third gonadotropin-releasing hormone from lamprey brain. Endocrinology (1993) 132(3):1125–31. 10.1210/endo.132.3.8440174 8440174

[11] Neill JD Duck LW Sellers JC Musgrove LC. A gonadotropin-releasing hormone (GnRH) receptor specific for GnRH II in primates. Biochem Biophys Res Commun (2001) 282(4):1012–8. 10.1006/bbrc.2001.4678 11352653

[12] Miyamoto K Hasegawa Y Nomura M Igarashi M Kangawa K Matsuo H. Identification of the second gonadotropin-releasing hormone in chicken hypothalamus: evidence that gonadotropin secretion is probably controlled by two distinct gonadotropin-releasing hormones in avian species. Proc Natl Acad Sci U S A (1984) 81(12):3874–8. 10.1073/pnas.81.12.3874 6427779 PMC345324

[13] King JA Mehl AE Tyndale-Biscoe CH Hinds L Millar RP. A second form of gonadotropin-releasing hormone (GnRH), with chicken GnRH II-like properties, occurs together with mammalian GnRH in marsupial brains. Endocrinology (1989) 125(5):2244–52. 10.1210/endo-125-5-2244 2676480

[14] Sealfon SC Weinstein H Millar RP Molecular mechanisms of ligand interaction with the gonadotropin-releasing hormone receptor. Endocr Rev (1997) 18(2):180–205. 10.1210/edrv.18.2.0295 9101136

[15] Fernald RD White RB. Gonadotropin-releasing hormone genes: phylogeny, structure, and functions. Front Neuroendocrinol (1999) 20(3):224–40. 10.1006/frne.1999.0181 10433863

[16] Millar RP. GnRH II and type II GnRH receptors. Trends Endocrinol Metab (2003) 14(1):35–43. 10.1016/S1043-2760(02)00016-4 12475610

[17] Millar RP King JA Structural and functional evolution of gonadotropin-releasing hormone. Int Rev Cytol (1987) 106:149–82. 10.1016/S0074-7696(08)61712-X 3294716

[18] Millar RP Lu ZL Pawson AJ Flanagan CA Morgan K Maudsley SR. Gonadotropin-releasing hormone receptors. Endocr Rev (2004) 25(2):235–75. 10.1210/er.2003-0002 15082521

[19] Millar RP Pawson AJ Morgan K Rissman EF Lu ZL. Diversity of actions of GnRHs mediated by ligand-induced selective signaling. Front Neuroendocrinol (2008) 29(1):17–35. 10.1016/j.yfrne.2007.06.002 17976709 PMC2667102

[20] White RB Eisen JA Kasten TL Fernald RD. Second gene for gonadotropin-releasing hormone in humans. Proc Natl Acad Sci U S A (1998) 95(1):305–9. 10.1073/pnas.95.1.305 9419371 PMC18209

[21] Stewart AJ Katz AA Millar RP Morgan K. Retention and silencing of prepro-GnRH-II and type II GnRH receptor genes in mammals. Neuroendocrinology (2009) 90(4):416–32. 10.1159/000233303 19657181

[22] Morgan K Millar RP. Evolution of GnRH ligand precursors and GnRH receptors in protochordate and vertebrate species. Gen Comp Endocrinol (2004) 139(3):191–7. 10.1016/j.ygcen.2004.09.015 15560865

[23] Xing J Wang C Kimura H Takasaki Y Kunimoto S Yoshimi A Resequencing and association analysis of PTPRA, a possible susceptibility gene for schizophrenia and autism spectrum disorders. PLoS One (2014) 9(11):e112531. 10.1371/journal.pone.0112531 25393624 PMC4231042

[24] Kenmochi N Suzuki T Uechi T Magoori M Kuniba M Higa S The human mitochondrial ribosomal protein genes: mapping of 54 genes to the chromosomes and implications for human disorders. Genomics (2001) 77(1–2):65–70. 10.1006/geno.2001.6622 11543634

[25] Sherwood NM Lovejoy DA Coe IR Origin of mammalian gonadotropin-releasing hormones. Endocr Rev (1993) 14(2):241–54. 10.1210/edrv-14-2-241 8325254

[26] Morgan K Sellar R Pawson AJ Lu ZL Millar RP. Bovine and ovine gonadotropin-releasing hormone (GnRH)-II ligand precursors and type II GnRH receptor genes are functionally inactivated. Endocrinology (2006) 147(11):5041–51. 10.1210/en.2006-0222 16916952

[27] Gestrin ED White RB Fernald RD. Second form of gonadotropin-releasing hormone in mouse: immunocytochemistry reveals hippocampal and periventricular distribution. FEBS Lett (1999) 448(2–3):289–91. 10.1016/S0014-5793(99)00361-0 10218494

[28] Chen A Yahalom D Ben-Aroya N Kaganovsky E Okon E Koch Y. A second isoform of gonadotropin-releasing hormone is present in the brain of human and rodents. FEBS Lett (1998) 435(2–3):199–203. 10.1016/S0014-5793(98)01064-3 9762908

[29] Millar R Lowe S Conklin D Pawson A Maudsley S Troskie B A novel mammalian receptor for the evolutionarily conserved type II GnRH. Proc Natl Acad Sci U S A (2001) 98(17):9636–41. 10.1073/pnas.141048498 11493674 PMC55504

[30] Pawson AJ Morgan K Maudsley SR Millar RP. Type II gonadotrophin-releasing hormone (GnRH-II) in reproductive biology. Reproduction (2003) 126(3):271–8. 10.1530/rep.0.1260271 12968935

[31] Temple JL Millar RP Rissman EF. An evolutionarily conserved form of gonadotropin-releasing hormone coordinates energy and reproductive behavior. Endocrinology (2003) 144(1):13–9. 10.1210/en.2002-220883 12488325

[32] Kauffman AS Wills A Millar RP Rissman EF. Evidence that the type-2 gonadotrophin-releasing hormone (GnRH) receptor mediates the behavioural effects of GnRH-II on feeding and reproduction in musk shrews. J Neuroendocrinol (2005) 17(8):489–97. 10.1111/j.1365-2826.2005.01334.x 16011485

[33] Rissman EF Alones VE Craig-Veit CB Millam JR. Distribution of chicken-II gonadotropin-releasing hormone in mammalian brain. J Comp Neurol (1995) 357(4):524–31. 10.1002/cne.903570404 7673483

[34] Desaulniers AT Cederberg RA Mills GA Lents CA White BR A putative role for GnRH-II and its receptor in spermatogenic function of boars. Proceedings of the Society for the Study of Reproduction 48th Annual Meeting San Juan, PR (2015). Abstract 530 p.

[35] Desaulniers AT Cederberg RA Mills GA Ford JJ Lents CA White BR LH-independent testosterone secretion is mediated by the interaction between GnRH2 and its receptor within porcine testes. Biol Reprod (2015) 93(2):45 10.1095/biolreprod.115.128082 26134865

[36] Urbanski HF White RB Fernald RD Kohama SG Garyfallou VT Densmore VS. Regional expression of mRNA encoding a second form of gonadotropin-releasing hormone in the macaque brain. Endocrinology (1999) 140(4):1945–8. 10.1210/endo.140.4.6779 10098535

[37] Lescheid DW Terasawa E Abler LA Urbanski HF Warby CM Millar RP A second form of gonadotropin-releasing hormone (GnRH) with characteristics of chicken GnRH-II is present in the primate brain. Endocrinology (1997) 138(12):5618–29. 10.1210/endo.138.12.5592 9389550

[38] Chen A Kaganovsky E Rahimipour S Ben-Aroya N Okon E Koch Y Two forms of gonadotropin-releasing hormone (GnRH) are expressed in human breast tissue and overexpressed in breast cancer: a putative mechanism for the antiproliferative effect of GnRH by down-regulation of acidic ribosomal phosphoproteins P1 and P2. Cancer Res (2002) 62(4):1036–44. 11861379

[39] Chen A Ganor Y Rahimipour S Ben-Aroya N Koch Y Levite M The neuropeptides GnRH-II and GnRH-I are produced by human T cells and trigger laminin receptor gene expression, adhesion, chemotaxis and homing to specific organs. Nat Med (2002) 8(12):1421–6. 10.1038/nm1202-801 12447356

[40] Choi KC Auersperg N Leung PC. Expression and antiproliferative effect of a second form of gonadotropin-releasing hormone in normal and neoplastic ovarian surface epithelial cells. J Clin Endocrinol Metab (2001) 86(10):5075–8. 10.1210/jcem.86.10.8100 11600588

[41] Choi JH Gilks CB Auersperg N Leung PC Immunolocalization of gonadotropin-releasing hormone (GnRH)-I, GnRH-II, and type I GnRH receptor during follicular development in the human ovary. J Clin Endocrinol Metab (2006) 91(11):4562–70. 10.1210/jc.2006-1147 16954155

[42] Chen A Zi K Laskar-Levy O Koch Y The transcription of the hGnRH-I and hGnRH-II genes in human neuronal cells is differentially regulated by estrogen. J Mol Neurosci (2002) 18(1–2):67–76. 10.1385/JMN:18:1-2:65 11931351

[43] Sand E Bergvall M Ekblad E D’Amato M Ohlsson B. Expression and distribution of GnRH, LH, and FSH and their receptors in gastrointestinal tract of man and rat. Regul Pept (2013) 187:24–8. 10.1016/j.regpep.2013.09.002 24103690

[44] Parker JD Malik M Catherino WH. Human myometrium and leiomyomas express gonadotropin-releasing hormone 2 and gonadotropin-releasing hormone 2 receptor. Fertil Steril (2007) 88(1):39–46. 10.1016/j.fertnstert.2006.11.098 17296196

[45] Desaulniers AT The Role of GnRH-II and Its Receptor in Testicular Function. Master’s thesis, University of Nebraska-Lincoln, Lincoln, NE (2013).

[46] van Biljon W Wykes S Scherer S Krawetz SA Hapgood J. Type II gonadotropin-releasing hormone receptor transcripts in human sperm. Biol Reprod (2002) 67(6):1741–9. 10.1095/biolreprod.101.002808 12444048

[47] Choi JH Choi KC Auersperg N Leung PC Differential regulation of two forms of gonadotropin-releasing hormone messenger ribonucleic acid by gonadotropins in human immortalized ovarian surface epithelium and ovarian cancer cells. Endocr Relat Cancer (2006) 13(2):641–51. 10.1677/erc.1.01057 16728589

[48] Cederberg RA Brauer VM Kerl JG Wiarda JR White BR Characterization of the porcine type II GnRH receptor gene. Biol Reprod (2009) 81(Suppl 1):Abstract 371 10.1093/biolreprod/81.s1.371

[49] Enomoto M Endo D Kawashima S Park MK. Human type II GnRH receptor mediates effects of GnRH on cell proliferation. Zoolog Sci (2004) 21(7):763–70. 10.2108/zsj.21.763 15277720

[50] Maiti K Oh DY Moon JS Acharjee S Li JH Bai DG Differential effects of gonadotropin-releasing hormone (GnRH)-I and GnRH-II on prostate cancer cell signaling and death. J Clin Endocrinol Metab (2005) 90(7):4287–98. 10.1210/jc.2004-1894 15870130

[51] Grundker C Gunthert AR Millar RP Emons G. Expression of gonadotropin-releasing hormone II (GnRH-II) receptor in human endometrial and ovarian cancer cells and effects of GnRH-II on tumor cell proliferation. J Clin Endocrinol Metab (2002) 87(3):1427–30. 10.1210/jcem.87.3.8437 11889221

[52] Grundker C Schlotawa L Viereck V Eicke N Horst A Kairies B Antiproliferative effects of the GnRH antagonist cetrorelix and of GnRH-II on human endometrial and ovarian cancer cells are not mediated through the GnRH type I receptor. Eur J Endocrinol (2004) 151(1):141–9. 10.1530/eje.0.1510141 15248835

[53] Desaulniers AT Cederberg RA Mills GA Lents CA White BR. Production of a gonadotropin-releasing hormone 2 receptor knockdown (GNRHR2 KD) swine line. Transgenic Res (2017) 26:567–75. 10.1007/s11248-017-0023-4 28534229 PMC5504211

[54] Darby S Stockley J Khan MM Robson CN Leung HY Gnanapragasam VJ. Expression of GnRH type II is regulated by the androgen receptor in prostate cancer. Endocr Relat Cancer (2007) 14(3):613–24. 10.1677/ERC-07-0041 17914092

[55] Densmore VS Urbanski HF. Effect of 17beta-estradiol on hypothalamic GnRH-II gene expression in the female rhesus macaque. J Mol Endocrinol (2004) 33(1):145–53. 10.1677/jme.0.0330145 15291749

[56] Khosravi S Leung PC. Differential regulation of gonadotropin-releasing hormone (GnRH)I and GnRHII messenger ribonucleic acid by gonadal steroids in human granulosa luteal cells. J Clin Endocrinol Metab (2003) 88(2):663–72. 10.1210/jc.2002-020866 12574197

[57] An BS Choi JH Choi KC Leung PC. Differential role of progesterone receptor isoforms in the transcriptional regulation of human gonadotropin-releasing hormone I (GnRH I) receptor, GnRH I, and GnRH II. J Clin Endocrinol Metab (2005) 90(2):1106–13. 10.1210/jc.2004-0318 15562029

[58] Kang SK Tai CJ Nathwani PS Leung PC. Differential regulation of two forms of gonadotropin-releasing hormone messenger ribonucleic acid in human granulosa-luteal cells. Endocrinology (2001) 142(1):182–92. 10.1210/endo.142.2.7960 11145581

[59] Chen A Laskar-Levy O Ben-Aroya N Koch Y. Transcriptional regulation of the human GnRH II gene is mediated by a putative cAMP response element. Endocrinology (2001) 142(8):3483–92. 10.1210/endo.142.8.8302 11459794

[60] Siler-Khodr TM Grayson M Eddy CA. Action of gonadotropin-releasing hormone II on the baboon ovary. Biol Reprod (2003) 68(4):1150–6. 10.1095/biolreprod.102.003483 12606432

[61] Zhang L Leng Q Mixson AJ. Alteration in the IL-2 signal peptide affects secretion of proteins in vitro and in vivo. J Gene Med (2005) 7(3):354–65. 10.1002/jgm.677 15619290

[62] Srinivasan S Bunch DO Feng Y Rodriguiz RM Li M Ravenell RL Deficits in reproduction and pro-gonadotropin-releasing hormone processing in male Cpefat mice. Endocrinology (2004) 145(4):2023–34. 10.1210/en.2003-1442 14715715

[63] Clarke IJ Cummins JT Karsch FJ Seeburg PH Nikolics K. GnRH-associated peptide (GAP) is cosecreted with GnRH into the hypophyseal portal blood of ovariectomized sheep. Biochem Biophys Res Commun (1987) 143(2):665–71. 10.1016/0006-291X(87)91405-7 3551953

[64] Adelman JP Mason AJ Hayflick JS Seeburg PH. Isolation of the gene and hypothalamic cDNA for the common precursor of gonadotropin-releasing hormone and prolactin release-inhibiting factor in human and rat. Proc Natl Acad Sci U S A (1986) 83(1):179–83. 10.1073/pnas.83.1.179 2867548 PMC322815

[65] Nikolics K Mason AJ Szonyi E Ramachandran J Seeburg PH. A prolactin-inhibiting factor within the precursor for human gonadotropin-releasing hormone. Nature (1985) 316(6028):511–7. 10.1038/316511a0 2863757

[66] Kasten TL White SA Norton TT Bond CT Adelman JP Fernald RD. Characterization of two new preproGnRH mRNAs in the tree shrew: first direct evidence for mesencephalic GnRH gene expression in a placental mammal. Gen Comp Endocrinol (1996) 104(1):7–19. 10.1006/gcen.1996.0135 8921350

[67] Cheon KW Lee HS Parhar IS Kang IS. Expression of the second isoform of gonadotrophin-releasing hormone (GnRH-II) in human endometrium throughout the menstrual cycle. Mol Hum Reprod (2001) 7(5):447–52. 10.1093/molehr/7.5.447 11331667

[68] Pfleger KD Bogerd J Millar RP Conformational constraint of mammalian, chicken, and salmon GnRHs, but not GnRH II, enhances binding at mammalian and nonmammalian receptors: evidence for preconfiguration of GnRH II. Mol Endocrinol (2002) 16(9):2155–62. 10.1210/me.2002-0159 12198251

[69] Gault PM Maudsley S Lincoln GA Evidence that gonadotropin-releasing hormone II is not a physiological regulator of gonadotropin secretion in mammals. J Neuroendocrinol (2003) 15(9):831–9. 10.1046/j.1365-2826.2003.01065.x 12899677

[70] Siler-Khodr TM Grayson M. Action of chicken II GnRH on the human placenta. J Clin Endocrinol Metab (2001) 86(2):804–10. 10.1210/jc.86.2.804 11158050

[71] Tsai PS Licht P. In vivo GnRH responsiveness of LH secretion in the female turtle, *Trachemys scripta*, in relation to the reproductive stage. Gen Comp Endocrinol (1993) 90(3):328–37. 10.1006/gcen.1993.1088 8224759

[72] Licht P Tsai PS Sotowska-Brochocka J. The nature and distribution of gonadotropin-releasing hormones in brains and plasma of ranid frogs. Gen Comp Endocrinol (1994) 94(2):186–98. 10.1006/gcen.1994.1075 7926629

[73] Pimstone B Epstein S Hamilton SM LeRoith D Hendricks S. Metabolic clearance and plasma half disappearance time of exogenous gonadotropin releasing hormone in normal subjects and in patients with liver disease and chronic renal failure. J Clin Endocrinol Metab (1977) 44(2):356–60. 10.1210/jcem-44-2-356 320223

[74] Tensen C Okuzawa K Blomenröhr M Rebersi F Leurs R Bogerd J Distinct efficacies for two endogenous ligands on a single cognate gonadoliberin receptor. Eur J Biochem (1997) 243:1–2, 134–40. 10.1111/j.1432-1033.1997.0134a.x 9030732

[75] Brauer VM Wiarda-Bell JR Desaulniers AT Cederberg RA White BR Functional activity of the porcine Gnrhr2 gene promoter in testis-derived cells is partially conferred by nuclear factor-kappaB, specificity protein 1 and 3 (SP1/3) and overlapping early growth response 1/SP1/3 binding sites. Gene (2016) 587(2):137–46. 10.1016/j.gene.2016.04.052 27134031

[76] Morgan K Conklin D Pawson AJ Sellar R Ott TR Millar RP. A transcriptionally active human type II gonadotropin-releasing hormone receptor gene homolog overlaps two genes in the antisense orientation on chromosome 1q.12. Endocrinology (2003) 144(2):423–36. 10.1210/en.2002-220622 12538601

[77] Faurholm B Millar RP Katz AA. The genes encoding the type II gonadotropin-releasing hormone receptor and the ribonucleoprotein RBM8A in humans overlap in two genomic loci. Genomics (2001) 78(1–2):15–8. 10.1006/geno.2001.6650 11707068

[78] Salicioni AM Xi M Vanderveer LA Balsara B Testa JR Dunbrack RL Jr Identification and structural analysis of human RBM8A and RBM8B: two highly conserved RNA-binding motif proteins that interact with OVCA1, a candidate tumor suppressor. Genomics (2000) 69(1):54–62. 10.1006/geno.2000.6315 11013075

[79] Thoms S Erdmann R. Dynamin-related proteins and Pex11 proteins in peroxisome division and proliferation. FEBS J (2005) 272(20):5169–81. 10.1111/j.1742-4658.2005.04939.x 16218949

[80] Neill JD. GnRH and GnRH receptor genes in the human genome. Endocrinology (2002) 143(3):737–43. 10.1210/endo.143.3.8705 11861490

[81] Gault PM Morgan K Pawson AJ Millar RP Lincoln GA. Sheep exhibit novel variations in the organization of the mammalian type II gonadotropin-releasing hormone receptor gene. Endocrinology (2004) 145(5):2362–74. 10.1210/en.2003-1625 14749360

[82] Neill JD Musgrove LC Duck LW. Newly recognized GnRH receptors: function and relative role. Trends Endocrinol Metab (2004) 15(8):383–92. 10.1016/S1043-2760(04)00186-9 15380810

[83] Chou CS MacCalman CD Leung PC Differential effects of gonadotropin-releasing hormone I and II on the urokinase-type plasminogen activator/plasminogen activator inhibitor system in human decidual stromal cells in vitro. J Clin Endocrinol Metab (2003) 88(8):3806–15. 10.1210/jc.2002-021955 12915673

[84] Liu J Maccalman CD Wang YL Leung PC. Promotion of human trophoblasts invasion by gonadotropin-releasing hormone (GnRH) I and GnRH II via distinct signaling pathways. Mol Endocrinol (2009) 23(7):1014–21. 10.1210/me.2008-0451 19372239 PMC5419179

[85] Namy O Rousset JP Napthine S Brierley I. Reprogrammed genetic decoding in cellular gene expression. Mol Cell (2004) 13(2):157–68. 10.1016/S1097-2765(04)00031-0 14759362

[86] Kozak M. Downstream secondary structure facilitates recognition of initiator codons by eukaryotic ribosomes. Proc Natl Acad Sci U S A (1990) 87(21):8301–5. 10.1073/pnas.87.21.8301 2236042 PMC54943

[87] Kozak M. Recognition of AUG and alternative initiator codons is augmented by G in position +4 but is not generally affected by the nucleotides in positions +5 and +6. EMBO J (1997) 16(9):2482–92. 10.1093/emboj/16.9.2482 9171361 PMC1169848

[88] Bertram G Innes S Minella O Richardson J Stansfield I Endless possibilities: translation termination and stop codon recognition. Microbiology (2001) 147(Pt 2):255–69. 10.1099/00221287-147-2-255 11158343

[89] Robinson DN Cooley L. Examination of the function of two kelch proteins generated by stop codon suppression. Development (1997) 124(7):1405–17. 9118811 10.1242/dev.124.7.1405

[90] Gudermann T Kalkbrenner F Dippel E Laugwitz KL Schultz G Specificity and complexity of receptor-G-protein interaction. Adv Second Messenger Phosphoprotein Res (1997) 31:253–62. 10.1016/S1040-7952(97)80023-7 9344256

[91] Schoneberg T Liu J Wess J. Plasma membrane localization and functional rescue of truncated forms of a G protein-coupled receptor. J Biol Chem (1995) 270(30):18000–6. 10.1074/jbc.270.30.18000 7629108

[92] Grosse R Schoneberg T Schultz G Gudermann T. Inhibition of gonadotropin-releasing hormone receptor signaling by expression of a splice variant of the human receptor. Mol Endocrinol (1997) 11(9):1305–18. 10.1210/mend.11.9.9966 9259321

[93] Pawson AJ Maudsley S Morgan K Davidson L Naor Z Millar RP. Inhibition of human type i gonadotropin-releasing hormone receptor (GnRHR) function by expression of a human type II GnRHR gene fragment. Endocrinology (2005) 146(6):2639–49. 10.1210/en.2005-0133 15761034

[94] Clay CM Nelson SE Digregorio GB Campion CE Wiedemann AL Nett RJ. Cell-specific expression of the mouse gonadotropin-releasing hormone (GnRH) receptor gene is conferred by elements residing within 500 bp of proximal 5’ flanking region. Endocrine (1995) 3(8):615–22. 10.1007/BF02953028 21153141

[95] Pincas H Amoyel K Counis R Laverriere JN. Proximal cis-acting elements, including steroidogenic factor 1, mediate the efficiency of a distal enhancer in the promoter of the rat gonadotropin-releasing hormone receptor gene. Mol Endocrinol (2001) 15(2):319–37. 10.1210/mend.15.2.0593 11158337

[96] Ngan ES Cheng PK Leung PC Chow BK. Steroidogenic factor-1 interacts with a gonadotrope-specific element within the first exon of the human gonadotropin-releasing hormone receptor gene to mediate gonadotrope-specific expression. Endocrinology (1999) 140(6):2452–62. 10.1210/endo.140.6.6759 10342829

[97] Duval DL Farris AR Quirk CC Nett TM Hamernik DL Clay CM. Responsiveness of the ovine gonadotropin-releasing hormone receptor gene to estradiol and gonadotropin-releasing hormone is not detectable in vitro but is revealed in transgenic mice. Endocrinology (2000) 141(3):1001–10. 10.1210/endo.141.3.7391 10698176

[98] Cederberg RA Smith JE McDonald EA Lee C Perkins AR White BR. Activity of the porcine gonadotropin-releasing hormone receptor gene promoter is partially conferred by a distal gonadotrope specific element (GSE) within an upstream enhancing region, two proximal GSEs and a retinoid X receptor binding site. Reprod Biol Endocrinol (2015) 13:45. 10.1186/s12958-015-0033-0 25981521 PMC4461931

[99] Faurholm B Cochrane S Millar RR Katz AA. Gene structure and promoter functional analysis of the marmoset type II GnRH receptor. J Mol Endocrinol (2007) 39(2):91–104. 10.1677/JME-06-0064 17693609

[100] McArdle CA Franklin J Green L Hislop JN. Signalling, cycling and desensitisation of gonadotrophin-releasing hormone receptors. J Endocrinol (2002) 173(1):1–11. 10.1677/joe.0.1730001 11927379

[101] Heding A Vrecl M Bogerd J McGregor A Sellar R Taylor PL Gonadotropin-releasing hormone receptors with intracellular carboxyl-terminal tails undergo acute desensitization of total inositol phosphate production and exhibit accelerated internalization kinetics. J Biol Chem (1998) 273(19):11472–7. 10.1074/jbc.273.19.11472 9565559

[102] Madziva MT Mkhize NN Flanagan CA Katz AA The carboxy-terminal tail or the intracellular loop 3 is required for beta-arrestin-dependent internalization of a mammalian type II GnRH receptor. Mol Cell Endocrinol (2015) 411:187–97. 10.1016/j.mce.2015.04.029 25957085

[103] Ronacher K Matsiliza N Nkwanyana N Pawson AJ Adam T Flanagan CA Serine residues 338 and 339 in the carboxyl-terminal tail of the type II gonadotropin-releasing hormone receptor are critical for beta-arrestin-independent internalization. Endocrinology (2004) 145(10):4480–8. 10.1210/en.2004-0075 15205374

[104] Maiti K Li JH Wang AF Acharjee S Kim WP Im WB GnRH-II analogs for selective activation and inhibition of non-mammalian and type-II mammalian GnRH receptors. Mol Cells (2003) 16(2):173–9. 14651258

[105] Flanagan CA Zhou W Chi L Yuen T Rodic V Robertson D The functional microdomain in transmembrane helices 2 and 7 regulates expression, activation, and coupling pathways of the gonadotropin-releasing hormone receptor. J Biol Chem (1999) 274(41):28880–6. 10.1074/jbc.274.41.28880 10506131

[106] Troskie B Illing N Rumbak E Sun YM Hapgood J Sealfon S Identification of three putative GnRH receptor subtypes in vertebrates. Gen Comp Endocrinol (1998) 112(3):296–302. 10.1006/gcen.1998.7156 9843635

[107] Pawson AJ McNeilly AS. The pituitary effects of GnRH. Anim Reprod Sci (2005) 88(1–2):75–94. 10.1016/j.anireprosci.2005.05.010 15982836

[108] Cheng CK Leung PCK Molecular biology of gonadotropin-releasing hormone (GnRH)-I, GnRH-II, and their receptors in humans. Endocr Rev (2005) 26(2):283–306. 10.1210/er.2003-0039 15561800

[109] Neill JD Duck LW Musgrove LC Potential regulatory role for GnRH II in gonadotropin secretion: molecular characterization of a GnRH II receptor in the pig pituitary. Abstracts of the 32nd Annual Meeting of the Society for Neuroscience Orlando, FL (2002). p. Abstract 1–97.

[110] Neill JD Duck LW Musgrove LC GnRH II receptor is encoded in genomes of human, monkey, and pig but not mouse. Abstracts of the 84th Annual Meeting of the Endocrine Society San Francisco, CA (2002). Abstract 74.9 p.

[111] Sanchez C Escrieut C Clerc P Gigoux V Waser B Reubi JC Characterization of a novel five-transmembrane domain cholecystokinin-2 receptor splice variant identified in human tumors. Mol Cell Endocrinol (2012) 349(2):170–9. 10.1016/j.mce.2011.10.010 22040601

[112] Ling K Wang P Zhao J Wu YL Cheng ZJ Wu GX Five-transmembrane domains appear sufficient for a G protein-coupled receptor: functional five-transmembrane domain chemokine receptors. Proc Natl Acad Sci U S A (1999) 96(14):7922–7. 10.1073/pnas.96.14.7922 10393923 PMC22163

[113] Perron A Sarret P Gendron L Stroh T Beaudet A. Identification and functional characterization of a 5-transmembrane domain variant isoform of the NTS2 neurotensin receptor in rat central nervous system. J Biol Chem (2005) 280(11):10219–27. 10.1074/jbc.M410557200 15637074

[114] Bokaei PB Ma XZ Byczynski B Keller J Sakac D Fahim S Identification and characterization of five-transmembrane isoforms of human vasoactive intestinal peptide and pituitary adenylate cyclase-activating polypeptide receptors. Genomics (2006) 88(6):791–800. 10.1016/j.ygeno.2006.07.008 16934434

[115] Wang AF Li JH Maiti K Kim WP Kang HM Seong JY Preferential ligand selectivity of the monkey type-II gonadotropin-releasing hormone (GnRH) receptor for GnRH-2 and its analogs. Mol Cell Endocrinol (2003) 209(1–2):33–42. 10.1016/j.mce.2003.08.004 14604814

[116] Kang SK Tai CJ Cheng KW Leung PC. Gonadotropin-releasing hormone activates mitogen-activated protein kinase in human ovarian and placental cells. Mol Cell Endocrinol (2000) 170(1–2):143–51. 10.1016/S0303-7207(00)00320-8 11162898

[117] Kim KY Choi KC Park SH Chou CS Auersperg N Leung PC. Type II gonadotropin-releasing hormone stimulates p38 mitogen-activated protein kinase and apoptosis in ovarian cancer cells. J Clin Endocrinol Metab (2004) 89(6):3020–6. 10.1210/jc.2003-031871 15181093

[118] Kim KY Choi KC Auersperg N Leung PC. Mechanism of gonadotropin-releasing hormone (GnRH)-I and -II-induced cell growth inhibition in ovarian cancer cells: role of the GnRH-I receptor and protein kinase C pathway. Endocr Relat Cancer (2006) 13(1):211–20. 10.1677/erc.1.01033 16601289

[119] Kim KY Choi KC Park SH Auersperg N Leung PC Extracellular signal-regulated protein kinase, but not c-Jun N-terminal kinase, is activated by type II gonadotropin-releasing hormone involved in the inhibition of ovarian cancer cell proliferation. J Clin Endocrinol Metab (2005) 90(3):1670–7. 10.1210/jc.2004-1636 15598681

[120] Eicke N Gunthert AR Emons G Grundker C. GnRH-II agonist [D-Lys6]GnRH-II inhibits the EGF-induced mitogenic signal transduction in human endometrial and ovarian cancer cells. Int J Oncol (2006) 29(5):1223–9. 17016655

[121] Yu WH Karanth S Walczewska A Sower SA McCann SM. A hypothalamic follicle-stimulating hormone-releasing decapeptide in the rat. Proc Natl Acad Sci U S A (1997) 94(17):9499–503. 10.1073/pnas.94.17.9499 9256511 PMC23238

[122] Mizunuma H Samson WK Lumpkin MD McCann SM. Evidence for an FSH-releasing factor in the posterior portion of the rat median eminence. Life Sci (1983) 33(20):2003–9. 10.1016/0024-3205(83)90739-7 6417428

[123] Padmanabhan V McNeilly AS. Is there an FSH-releasing factor? Reproduction (2001) 121(1):21–30. 10.1530/rep.0.1210021 11226026

[124] Latimer VS Rodrigues SM Garyfallou VT Kohama SG White RB Fernald RD Two molecular forms of gonadotropin-releasing hormone (GnRH-I and GnRH-II) are expressed by two separate populations of cells in the rhesus macaque hypothalamus. Brain Res Mol Brain Res (2000) 75(2):287–92. 10.1016/S0169-328X(99)00316-2 10686350

[125] Okada Y Murota-Kawano A Kakar SS Winters SJ. Evidence that gonadotropin-releasing hormone (GnRH) II stimulates luteinizing hormone and follicle-stimulating hormone secretion from monkey pituitary cultures by activating the GnRH I receptor. Biol Reprod (2003) 69(4):1356–61. 10.1095/biolreprod.103.016162 12801988

[126] Millar RP Milton RC Follett BK King JA. Receptor binding and gonadotropin-releasing activity of a novel chicken gonadotropin-releasing hormone ([His5, Trp7, Tyr8]GnRH) and a D-Arg6 analog. Endocrinology (1986) 119(1):224–31. 10.1210/endo-119-1-224 3013586

[127] Balik A Jindrichova M Bhattacharyya S Zemkova H. GnRH-I and GnRH-II-induced calcium signaling and hormone secretion in neonatal rat gonadotrophs. Physiol Res (2009) 58(5):709–16. 19093727 10.33549/physiolres.931632

[128] Densmore VS Urbanski HF. Relative effect of gonadotropin-releasing hormone (GnRH)-I and GnRH-II on gonadotropin release. J Clin Endocrinol Metab (2003) 88(5):2126–34. 10.1210/jc.2002-021359 12727965

[129] Urbanski HF. Selective targeting of GnRH-II neurons to block ovulation. Contraception (2015) 91(5):423–5. 10.1016/j.contraception.2014.09.010 25444718 PMC4377115

[130] Bowen A Khan S Berghman L Kirby JD Wettemann RP Vizcarra JA. Immunization of pigs against chicken gonadotropin-releasing hormone-II and lamprey gonadotropin-releasing hormone-III: effects on gonadotropin secretion and testicular function. J Anim Sci (2006) 84(11):2990–9. 10.2527/jas.2006-235 17032793

[131] Millar RP Gonadotropin-releasing hormones and their receptors. In: Fauser BCM, editor. Reproductive Medicine: Molecular Cellular and Genetic Fundamentals. Lancaster, UK: Parthenon Publishing (2002). p. 199–224.

[132] Maney DL Richardson RD Wingfield JC. Central administration of chicken gonadotropin-releasing hormone-II enhances courtship behavior in a female sparrow. Horm Behav (1997) 32(1):11–8. 10.1006/hbeh.1997.1399 9344687

[133] Kauffman AS Bojkowska K Wills A Rissman EF. Gonadotropin-releasing hormone-II messenger ribonucleic acid and protein content in the mammalian brain are modulated by food intake. Endocrinology (2006) 147(11):5069–77. 10.1210/en.2006-0615 16873537

[134] Kauffman AS Rissman EF A critical role for the evolutionarily conserved gonadotropin-releasing hormone II: mediation of energy status and female sexual behavior. Endocrinology (2004) 145(8):3639–46. 10.1210/en.2004-0148 15105381

[135] Schneider JS Rissman EF. Gonadotropin-releasing hormone II: a multi-purpose neuropeptide. Integr Comp Biol (2008) 48(5):588–95. 10.1093/icb/icn018 21669818 PMC6283013

[136] Barnett DK Bunnell TM Millar RP Abbott DH. Gonadotropin-releasing hormone II stimulates female sexual behavior in marmoset monkeys. Endocrinology (2006) 147(1):615–23. 10.1210/en.2005-0662 16179411

[137] Kendrick KM Dixson AF. Luteinizing hormone releasing hormone enhances proceptivity in a primate. Neuroendocrinology (1985) 41(6):449–53. 10.1159/000124218 3935943

[138] Kauffman AS Rissman EF The evolutionarily conserved gonadotropin-releasing hormone II modifies food intake. Endocrinology (2004) 145(2):686–91. 10.1210/en.2004-0148 14576176

[139] Hsueh AJ Erickson GF Extra-pituitary inhibition of testicular function by luteinising hormone releasing hormone. Nature (1979) 281(5726):66–7. 10.1038/281066a0 233123

[140] Clayton RN Katikineni M Chan V Dufau ML Catt KJ. Direct inhibition of testicular function by gonadotropin-releasing hormone: mediation by specific gonadotropin-releasing hormone receptors in interstitial cells. Proc Natl Acad Sci U S A (1980) 77(8):4459–63. 10.1073/pnas.77.8.4459 6254027 PMC349863

[141] Bahk JY Hyun JS Chung SH Lee H Kim MO Lee BH Stage specific identification of the expression of GnRH mRNA and localization of the GnRH receptor in mature rat and adult human testis. J Urol (1995) 154(5):1958–61. 10.1097/00005392-199511000-00105 7563392

[142] Bull P Morales P Huyser C Socias T Castellon EA. Expression of GnRH receptor in mouse and rat testicular germ cells. Mol Hum Reprod (2000) 6(7):582–6. 10.1093/molehr/6.7.582 10871643

[143] Zerani M Catone G Quassinti L Maccari E Bramucci M Gobbetti A In vitro effects of gonadotropin-releasing hormone (GnRH) on Leydig cells of adult alpaca (*Lama pacos*) testis: GnRH receptor immunolocalization, testosterone and prostaglandin synthesis, and cyclooxygenase activities. Domest Anim Endocrinol (2011) 40(1):51–9. 10.1016/j.domaniend.2010.08.006 20961724

[144] Sharpe RM Fraser HM. HCG stimulation of testicular LHRH-like activity. Nature (1980) 287(5783):642–3. 10.1038/287642a0 6253804

[145] Sharpe RM Doogan DG Cooper I Direct effects of a luteinizing hormone-releasing hormone agonist on intratesticular levels of testosterone and interstitial fluid formation in intact male rats. Endocrinology (1983) 113(4):1306–13. 10.1210/endo-113-4-1306 6413194

[146] Sharpe RM Cooper I. Stimulatory effect of LHRH and its agonists on Leydig cell steroidogenesis in vitro. Mol Cell Endocrinol (1982) 26(1–2):141–50. 10.1016/0303-7207(82)90012-0 6177569

[147] Browning JY D’Agata R Steinberger A Grotjan HE Jr Steinberger E. Biphasic effect of gonadotropin-releasing hormone and its agonist analog (HOE766) on in vitro testosterone production by purified rat Leydig cells. Endocrinology (1983) 113(3):985–91. 10.1210/endo-113-3-985 6409594

[148] Molcho J Eli Y Zakut H Naor Z. Stimulation of prostaglandin E and testosterone production in rat interstitial cells by a gonadotropin-releasing hormone agonist. Endocrinology (1984) 114(6):2382–7. 10.1210/endo-114-3-1048 6373242

[149] McGuire NL Bentley GE. Neuropeptides in the gonads: from evolution to pharmacology. Front Pharmacol (2010) 1:114. 10.3389/fphar.2010.00114 21607065 PMC3095369

[150] Kah O Lethimonier C Lareyre JJ Gonadotrophin-releasing hormone (GnRH) in the animal kingdom. J Soc Biol (2004) 198(1):53–60. 10.1051/jbio/2004198010053 15146956

[151] Pazos AJ Mathieu M. Effects of five natural gonadotropin-releasing hormones on cell suspensions of marine bivalve gonad: stimulation of gonial DNA synthesis. Gen Comp Endocrinol (1999) 113(1):112–20. 10.1006/gcen.1998.7186 9882550

[152] Adams BA Tello JA Erchegyi J Warby C Hong DJ Akinsanya KO Six novel gonadotropin-releasing hormones are encoded as triplets on each of two genes in the protochordate, *Ciona intestinalis*. Endocrinology (2003) 144(5):1907–19. 10.1210/en.2002-0216 12697698

[153] Di Fiore MM Rastogi RK Ceciliani F Messi E Botte V Botte L Mammalian and chicken I forms of gonadotropin-releasing hormone in the gonads of a protochordate, *Ciona intestinalis*. Proc Natl Acad Sci U S A (2000) 97(5):2343–8. 10.1073/pnas.040549097 10688887 PMC15803

[154] Lin YM Poon SL Choi JH Lin JS Leung PC Huang BM. Transcripts of testicular gonadotropin-releasing hormone, steroidogenic enzymes, and intratesticular testosterone levels in infertile men. Fertil Steril (2008) 90(5):1761–8. 10.1016/j.fertnstert.2007.08.078 18082732

[155] Ma C Song H Guan K Zhou J Xia X Li F. Characterization of swine testicular cell line as immature porcine Sertoli cell line. In Vitro Cell Dev Biol Anim (2016) 52(4):427–33. 10.1007/s11626-015-9994-8 26744029

[156] Khan MA Prevost M Waterston MM Harvey MJ Ferro VA. Effect of immunisation against gonadotrophin releasing hormone isoforms (mammalian GnRH-I, chicken GnRH-II and lamprey GnRH-III) on murine spermatogenesis. Vaccine (2007) 25(11):2051–63. 10.1016/j.vaccine.2006.11.038 17240004

[157] Wise T Zanella EL Lunstra DD Ford JJ. Relationships of gonadotropins, testosterone, and cortisol in response to GnRH and GnRH antagonist in boars selected for high and low follicle-stimulating hormone levels. J Anim Sci (2000) 78(6):1577–90. 10.2527/2000.7861577x 10875642

[158] Zanella EL Lunstra DD Wise TH Kinder JE Ford JJ. GnRH antagonist inhibition of gonadotropin and steroid secretion in boars in vivo and steroid production in vitro. J Anim Sci (2000) 78(6):1591–7. 10.2527/2000.7861591x 10875643

[159] Lents CA Thorson JF Desaulniers AT White BR. RFamide-related peptide 3 and gonadotropin-releasing hormone-II are autocrine-paracrine regulators of testicular function in the boar. Mol Reprod Dev (2017) 84:994–1003. 10.1002/mrd.22830 28475264

[160] Chou CS Beristain AG MacCalman CD Leung PC. Cellular localization of gonadotropin-releasing hormone (GnRH) I and GnRH II in first-trimester human placenta and decidua. J Clin Endocrinol Metab (2004) 89(3):1459–66. 10.1210/jc.2003-031636 15001648

[161] Islami D Bischof P Chardonnens D. Possible interactions between leptin, gonadotrophin-releasing hormone (GnRH-I and II) and human chorionic gonadotrophin (hCG). Eur J Obstet Gynecol Reprod Biol (2003) 110(2):169–75. 10.1016/S0301-2115(03)00185-4 12969578

[162] Islami D Chardonnens D Campana A Bischof P. Comparison of the effects of GnRH-I and GnRH-II on HCG synthesis and secretion by first trimester trophoblast. Mol Hum Reprod (2001) 7(1):3–9. 10.1093/molehr/7.1.3 11134354

[163] Eicke N Gunthert AR Viereck V Siebold D Behe M Becker T GnRH-II receptor-like antigenicity in human placenta and in cancers of the human reproductive organs. Eur J Endocrinol (2005) 153(4):605–12. 10.1530/eje.1.02005 16189182

[164] Liu J Cao B Li YX Wu XQ Wang YL. GnRH I and II up-regulate MMP-26 expression through the JNK pathway in human cytotrophoblasts. Reprod Biol Endocrinol (2010) 8:5. 10.1186/1477-7827-8-5 20074375 PMC2819245

[165] Chou CS Zhu H MacCalman CD Leung PC Regulatory effects of gonadotropin-releasing hormone (GnRH) I and GnRH II on the levels of matrix metalloproteinase (MMP)-2, MMP-9, and tissue inhibitor of metalloproteinases-1 in primary cultures of human extravillous cytotrophoblasts. J Clin Endocrinol Metab (2003) 88(10):4781–90. 10.1210/jc.2003-030659 14557455

[166] Chou CS Zhu H Shalev E MacCalman CD Leung PC. The effects of gonadotropin-releasing hormone (GnRH) I and GnRH II on the urokinase-type plasminogen activator/plasminogen activator inhibitor system in human extravillous cytotrophoblasts in vitro. J Clin Endocrinol Metab (2002) 87(12):5594–603. 10.1210/jc.2002-020883 12466358

[167] Emons G Schroder B Ortmann O Westphalen S Schulz KD Schally AV. High affinity binding and direct antiproliferative effects of luteinizing hormone-releasing hormone analogs in human endometrial cancer cell lines. J Clin Endocrinol Metab (1993) 77(6):1458–64. 10.1210/jc.77.6.1458 8263128

[168] Siler-Khodr TM Yu FQ Wei P Tao SX Liu YX. Contraceptive action of a gonadotropin-releasing hormone II analog in the rhesus monkey. J Clin Endocrinol Metab (2004) 89(9):4513–20. 10.1210/jc.2004-032087 15356056

[169] Siler-Khodr TM Yu FQ Wei P Tao SX Coulhart S Mactyszczyk S Dose-related actions of GnRH II analog in the cycling rhesus monkey. Contraception (2006) 74(2):157–64. 10.1016/j.contraception.2005.12.013 16860054

[170] Grundker C Emons G. Role of gonadotropin-releasing hormone (GnRH) in ovarian cancer. Reprod Biol Endocrinol (2003) 1:65. 10.1186/1477-7827-1-65 14594454 PMC239893

[171] Limonta P Moretti RM Montagnani Marelli M Motta M. The biology of gonadotropin hormone-releasing hormone: role in the control of tumor growth and progression in humans. Front Neuroendocrinol (2003) 24(4):279–95. 10.1016/j.yfrne.2003.10.003 14726258

[172] Park S Han JM Cheon J Hwang JI Seong JY. Apoptotic death of prostate cancer cells by a gonadotropin-releasing hormone-II antagonist. PLoS One (2014) 9(6):e99723. 10.1371/journal.pone.0099723 24926857 PMC4057422

[173] Ling Poon S Lau MT Hammond GL Leung PC. Gonadotropin-releasing hormone-II increases membrane type I metalloproteinase production via beta-catenin signaling in ovarian cancer cells. Endocrinology (2011) 152(3):764–72. 10.1210/en.2010-0942 21239435

[174] Skog J Wurdinger T van Rijn S Meijer DH Gainche L Sena-Esteves M Glioblastoma microvesicles transport RNA and proteins that promote tumour growth and provide diagnostic biomarkers. Nat Cell Biol (2008) 10(12):1470–6. 10.1038/ncb1800 19011622 PMC3423894

[175] Fister S Gunthert AR Emons G Grundker C. Gonadotropin-releasing hormone type II antagonists induce apoptotic cell death in human endometrial and ovarian cancer cells in vitro and in vivo. Cancer Res (2007) 67(4):1750–6. 10.1158/0008-5472.CAN-06-3222 17308117

[176] Hong IS Klausen C Cheung AP Leung PC. Gonadotropin-releasing hormone-I or -II interacts with IGF-I/Akt but not connexin 43 in human granulosa cell apoptosis. J Clin Endocrinol Metab (2012) 97(2):525–34. 10.1210/jc.2011-1229 22112812

[177] Grundker C Fost C Fister S Nolte N Gunthert AR Emons G. Gonadotropin-releasing hormone type II antagonist induces apoptosis in MCF-7 and triple-negative MDA-MB-231 human breast cancer cells in vitro and in vivo. Breast Cancer Res (2010) 12(4):R49. 10.1186/bcr2606 20630060 PMC2949636

[178] Kim DK Yang JS Maiti K Hwang JI Kim K Seen D A gonadotropin-releasing hormone-II antagonist induces autophagy of prostate cancer cells. Cancer Res (2009) 69(3):923–31. 10.1158/0008-5472.CAN-08-2115 19176390

